# Experimental priming of independent and interdependent activity does not affect culturally variable psychological processes

**DOI:** 10.1098/rsos.161025

**Published:** 2017-05-17

**Authors:** Kesson Magid, Vera Sarkol, Alex Mesoudi

**Affiliations:** 1 Department of Anthropology, Durham University, Durham, UK; 2 School of Biological and Chemical Sciences, Queen Mary University of London, London, UK; 3 Human Biological and Cultural Evolution Group, Department of Biosciences, University of Exeter, Penryn, UK

**Keywords:** cultural cognition, cultural evolution, cultural psychology, ecocultural hypothesis, priming, self-construal

## Abstract

Cultural psychologists have shown that people from Western countries exhibit more independent self-construal and analytic (rule-based) cognition than people from East Asia, who exhibit more interdependent self-construal and holistic (relationship-based) cognition. One explanation for this cross-cultural variation is the ecocultural hypothesis, which links contemporary psychological differences to ancestral differences in subsistence and societal cohesion: Western thinking formed in response to solitary herding, which fostered independence, while East Asian thinking emerged in response to communal rice farming, which fostered interdependence. Here, we report two experiments that tested the ecocultural hypothesis in the laboratory. In both, participants played one of two tasks designed to recreate the key factors of working alone and working together. Before and after each task, participants completed psychological measures of independent–interdependent self-construal and analytic–holistic cognition. We found no convincing evidence that either solitary or collective tasks affected any of the measures in the predicted directions. This fails to support the ecocultural hypothesis. However, it may also be that our priming tasks are inappropriate or inadequate for simulating subsistence-related behavioural practices, or that these measures are fixed early in development and therefore not experimentally primable, despite many previous studies that have purported to find such priming effects.

## 1. Introduction

Cultural psychologists have documented hitherto-unsuspected cross-cultural variation in various aspects of human cognitive and social psychological processes [1–4]. For example, it was once thought on the basis of studies of Western participants that people are universally biased towards explaining others' actions in terms of intrinsic personality traits such as laziness or conscientiousness (‘dispositional social attribution’) [5]. Yet subsequent research found that East Asian participants are more likely to endorse explanations in terms of extrinsic situations, such as task demands or social norms (‘situational social attribution’) [6]. Early studies with Western participants suggested that people showed self-enhancement bias, rating themselves unrealistically highly on various desirable characteristics such as intelligence, attractiveness and health [7]. Yet, subsequent studies with East Asian participants found this self-enhancement bias to be much reduced, and often entirely absent [8,9]. Cognition, too, shows systematic cross-cultural variation in domains such as categorization and reasoning, where Western participants typically use formal, rule-based reasoning, while East Asian participants typically use intuitive, relationship-based reasoning [10,11]. For example, while Westerners group objects according to formal rules (e.g. grouping ‘cow’ and ‘horse’ together as examples of the formal category of farmyard animals), East Asians group objects according to the relationships between them (e.g. grouping ‘horse’ and ‘saddle’ together because horses wear saddles) [12].

More recent research in cultural psychology has revealed many nuances in these cross-cultural differences, but also repeated confirmation of the existence of meaningful cultural variation in psychological processes. For example, the aforementioned findings related to social attribution have been clarified by newer findings showing that, for example, (i) while dispositional attribution seems to be cross-culturally consistent, East Asians show greater endorsement of situational factors than Westerners only when situational information is made salient [13]; (ii) North Americans endorse dispositional explanations mostly for ordinary actions and instead use mental state attributions for puzzling or unusual actions [14]; and (iii) higher socio-economic status within both Western and non-Western countries is associated with more dispositional attribution [15]. Similarly, self-enhancement is thought by some to be a culturally universal underlying motivation which manifests itself in different ways cross-culturally: for example, Westerners self-enhance on traits such as originality or self-reliance while East Asians self-enhance on traits such as compromise and loyalty; modesty norms are stronger in East Asia than the West, causing East Asian people to suppress self-enhancing opinions in public settings [16]. In these and other cases, initial findings of dramatic cross-cultural differences have given way to more nuanced understandings of how cultural background interacts with contexts and situations to create culturally variable psychological responses.

Given these findings, researchers have attempted to summarize this variation in theoretically coherent dimensions or conceptual schemes. One concerns self-construal [17] or social orientation [18], where people with independent self-construal see themselves as separate and bounded from others and value individual goals and personal autonomy, and people with interdependent self-construal see themselves as overlapping in identity with others and value collective goals and harmony with others. Another dimension relates to cognition [11], where analytic cognition involves a focus on single objects or entities independent of their wider contexts, explaining events in terms of entities' internal traits, and the use of formal logic, while holistic cognition involves a focus on relationships between objects or entities within wider contexts, explaining events in terms of contexts or situations, and use of relationship-based reasoning. Countries and societies high in independent self-construal, such as the USA, also tend to be high in analytic cognition, and countries and societies high in interdependent self-construal, such as China, also tend to be high in holistic cognition [19]. These dimensions extend beyond a simplistic East–West dichotomy. For example, Russia is both more interdependent and more cognitively holistic than the USA [19], and Hokkaido is more independent than the rest of Japan [20].

Yet while there have been many demonstrations of interdependent–independent and holistic–analytic cross-cultural variation in psychological processes in contemporary Western and East Asian populations, it is less clear how this cultural variation originated. One leading theory is the *ecocultural hypothesis* [11,21], which links present-day psychological differences to historical differences in means of subsistence and working practices. Specifically, it is argued that Western thought originated in Ancient Greece and surrounding areas where the primary means of subsistence was herding. This largely solitary and mobile activity fostered self-reliance and independence from others, which in turn led to the independent and analytic thinking style seen today in Western Europe and European descendants living in its colonial offshoots (e.g. North America, Australasia). East Asian thought, on the other hand, arose in Ancient China where the main means of subsistence was farming, and in particular rice farming [22]. This more collaborative and sedentary activity requires intensive cooperation and coordination, which fostered mutual reliance and interdependence with others. This led to the interdependent and holistic thinking style seen today in East Asia, given China's long-lasting historical influence in the entire region. This ecocultural hypothesis is summarized nicely by Uskul *et al*.:

Farming requires harmonious group collaboration. Moreover, farmers are largely sedentary; they are tied to the land they cultivate and, thus, to fixed communities. These factors are likely to encourage a high degree of social interdependence. In contrast, herding activities do not require much cooperation, but rely on individual decision making and autonomy. Moreover, herders are much less sedentary; their capital can be moved to any location with enough nutrition for animals. Herding communities are therefore unlikely to exert much pressure toward cooperation or conformity. Instead, they foster individualistic or independent social orientations [21, p. 8552].

While plausible, the validity of such a hypothesis cannot rest purely on a verbal description of historical events, which can easily be cherry-picked. Consequently, researchers have sought to test the ecocultural hypothesis using contemporary cross-cultural comparisons. The best evidence in support of the ecocultural hypothesis comes from two natural contrasts. The first, Uskul *et al*. [21], found that members of a herding community in modern-day Turkey show more independent responses in perceptual, reasoning and categorization tasks than members of fishing and farming communities from the same ethnic, linguistic and educational background as the herders. The second, Talhelm *et al*. [22], refined the ecocultural hypothesis to examine different kinds of farming that vary in their activity patterns. People living in parts of China that have a long tradition of rice farming showed more interdependent responses in three tasks measuring object categorization, implicit individualism and loyalty/nepotism compared with people living in parts of China that have a tradition of wheat farming. The argument here is that rice farming is more labour-intensive than wheat farming, making it a more communal activity. Rice farming also requires the village-wide coordination and reciprocation of water usage in a way that wheat farming does not. Talhelm *et al*. describe the contrast thus:

Because rice paddies need standing water, people in rice regions build elaborate irrigation systems that require farmers to cooperate. In irrigation networks, one family's water use can affect their neighbors, so rice farmers have to coordinate their water use. Irrigation networks also require many hours each year to build, dredge, and drain—a burden that often falls on villages, not isolated individuals…. In comparison, wheat is easier to grow. Wheat does not need to be irrigated, so wheat farmers can rely on rainfall, which they do not coordinate with their neighbors. Planting and harvesting wheat certainly takes work, but only half as much as rice…. The lighter burden means farmers can look after their own plots without relying as much on their neighbors [22, p. 604].

While these studies are innovative and certainly provide suggestive evidence in support of the ecocultural hypothesis, they suffer from two potential problems that are inherent when studying naturally varying (i.e. non-randomly assigned) populations. First, these studies are, by design, correlational. While attempts were made to control for potential confounds, such as ensuring all participants came from the same ethnic background, it is possible that an unidentified confound generated the differences rather than subsistence-related activity. Second, there is ambiguity over the precise aspects of the subsistence practices that are driving differences in thinking styles. The quotes above variously discuss working together versus working alone, reliance on others versus reliance on oneself, active coordination and cooperation with others versus an absence of coordination and cooperation, and intensity or difficulty of the subsistence (e.g. wheat is easier to grow than rice). The problem with natural contrasts is that all of these differences co-occur, and it is unclear which are responsible for independent and interdependent thinking styles.

Here, we provide a further test of the ecocultural hypothesis, using an alternative method that can potentially resolve these issues: laboratory experiments. While obviously featuring lower external validity than comparisons of naturally occurring inter-group variation, laboratory experiments afford greater control over confounding variables and greater power to systematically manipulate causal factors—in this case relating to subsistence-related activity patterns. In two experiments, we recreated and manipulated some of those factors predicted to have generated cross-cultural differences in thinking styles according to the ecocultural hypothesis.

In both experiments, all participants completed various psychological measures of independent–interdependent thinking both before and after a task. Half of the participants in each experiment completed a solitary task where payoffs depend solely on one's own actions, with no coordination or cooperation with others, as is hypothesized to characterize herding. The other half of the participants completed a collective task where payoffs depend on one's own and others' actions, and which involve some kind of coordination or cooperation, as is hypothesized to characterize farming, and in particular rice farming. By necessity, our tasks do not map precisely onto real-life subsistence practices, which are complex and varied. But we think that our tasks capture at least some of the essential elements described in verbal descriptions of the ecocultural hypothesis, such as those quoted above.

Experiment 1 used computer-based tasks drawn from experimental economics: a solitary multi-armed bandit game, which is played alone and where payoffs are independent of others' decisions, and a collective public goods game, where payoffs depend on the entire group's contributions to a public pot. Experiment 2 used more intensive, face-to-face origami folding, which participants either did alone and were rewarded independently of others' performance (the solitary version), or did in a group and were rewarded based on the whole group's performance (the collective version). This addressed a potential criticism of Experiment 1 that computer-implemented public goods games, while cooperative in a technical sense, are not very intensely social given that participants are sat at separate computer terminals.

The prediction derived from the ecocultural hypothesis in both experiments is that the solitary condition should increase independent or analytic responses on the tasks, and the collective condition should increase interdependent or holistic responses, relative to the pre-task baseline. This pre-test/post-test design was used to control for known individual variation in independence–interdependence [23]. For the tasks, we chose a variety of measures of both interdependent–independent self-construal (e.g. pronoun use and questionnaire measures of individualism–collectivism) and non-social cognitive measures (e.g. categorization and drawing style), each of which has previously been shown to vary cross-culturally.

Our methodology is predicated on the assumption that independent–interdependent thinking is malleable and can be primed by immediate situations. This assumption is supported by many previous studies that have primed independent–interdependent thinking in the laboratory. A meta-analysis of 67 such priming studies [24] found an overall weighted mean effect size of *d* = 0.34, with most studies having *d* = 0.3–0.6, traditionally considered small to moderate effects. While this shows that cultural orientation can be primed experimentally, the majority of the primes used in these 67 studies are unsuitable for making inferences about the ecocultural hypothesis (or indeed any other hypothesis) for the origin of psychological variation, as they were not designed for this purpose. For example, most priming studies use pronoun-circling, where the independence prime involves circling independent pronouns (e.g. I, me or my) and the interdependence prime involves circling interdependent pronouns (e.g. us, we or our) [25]. It is unclear how verbal tasks such as these relate to the solitary versus collective activity patterns (e.g. herding, farming) intrinsic to the ecocultural hypothesis. Only one previous study to our knowledge has used solitary versus collective *activity* as a prime for independence–interdependence [26]. Here, participants who completed an anagram task in groups subsequently chose two dissimilar candy bars, while participants who completed the same task alone subsequently chose two identical candy bars. Choosing two different candy bars was interpreted as indicating a focus on the wider collective (by leaving a wider choice of candy for other group members), although this is a rather ambiguous and limited measure of independent–interdependent thinking. We instead used a battery of dependent measures, both verbal and non-verbal, that tap various aspects of independent–interdependent self-construal and analytic–holistic cognition.

To restate this methodological approach in a different way, we are in effect seeking to ‘simulate history’ within experimental microsocieties in the laboratory, in order to test causal hypotheses about the origin of present-day cultural variation that cannot be tested with historical analysis alone. This approach is not common, but it does have precedent in the interdisciplinary study of cultural evolution [27] where researchers have sought to recreate the historical emergence of hierarchical social organization [28], patterns of diversity in material artefacts observed in the archaeological record [29] and aspects of language structure [30]. On this view, effects observable in the laboratory during short time periods, even if small in their magnitude, can be magnified when repeated over historical time and in multiple real-life generations of people to generate meaningful large-scale cultural variation [31].

## 2. Experiment 1: economic games as primes

In Experiment 1, we used established computer-based tasks from experimental economics to simulate the activity: the multi-armed bandit game to simulate independent, solitary activity (analogous to herding), and the public goods game with punishment to simulate interdependent, collective activity (analogous to farming).

### 2.1. Material and methods

#### 2.1.1. Design

A 2 × 2 mixed design was used where the within-participant factor was priming (before versus after the priming task) and the between-participant factor was the activity condition (collective versus solitary activity). The dependent variables were the answers on seven measures obtained from five tasks (two tasks gave two measures each) before and after the priming task. It was predicted that participants in the collective condition would shift their answers towards greater interdependence or holistic cognition, while participants in the solitary condition would shift towards greater independence or analytic cognition. Statistically, this would be indicated by a significant interaction between activity condition (solitary versus collective) and time (before priming versus after priming).

#### 2.1.2. Materials: dependent measures

The five dependent measures were presented in a paper booklet. All five measures were administered both before and after the priming task, although with different items or in different combinations to avoid participants simply recalling their previous answers, and were counterbalanced in two randomly assigned versions of the questionnaires. Two tasks—social orientation and pronoun use—measured self-construal, with social orientation measured using self-report Likert responses and pronoun use providing a more implicit measure of self-construal. Three tasks—categorization, portrait selection and landscape drawing—measured cognitive style. Categorization was verbal, while portrait selection and landscape drawing were non-verbal. Using a range of verbal and non-verbal, explicit and implicit measures provides a range of opportunities for the priming task to affect thinking style.

First, a categorization measure was adapted from Markman & Hutchinson [32]. Participants were shown a target word (e.g. dog) and then asked which of two further words best group with the target: a word linked by taxonomic category (e.g. cat) would indicate analytic thinking, and a word linked by relationship (e.g. bone) would indicate holistic thinking. We also obtained a brief explanation of the participant's choice, to verify that the reasoning was indeed based on analytic or holistic thought (e.g. ‘dog’ may have been paired to ‘cat’ because of relations, e.g. ‘dogs chase cats’, in which case this would be counted as holistic rather than analytic). Ten word sets were presented before the prime, and 10 different sets after the prime.

Second, a portrait selection measure was adapted from Masuda *et al*. [33], where five sets of portrait pictures were manipulated to have a person appear in different sizes in a background. A more analytic response would be to select the portrait with a larger face-to-frame ratio, such that the face is the sole focal object of attention. Holistic responses would involve selecting the portrait with the smaller face-to-frame ratio and the greatest background information, placing the face within a larger context containing other objects.

Third, participants completed a landscape drawing task also adapted from Masuda *et al*. [33]. Participants were given an A4 sheet of paper with a black frame and asked to draw a landscape including either at least a barn, a tree, a cow, a road and a horizon, or at least a house, a tree, a river, a person and a horizon, with one set of instructions given before and the other after the priming task. Additional objects were also permitted. Based on cross-cultural comparisons [33,34], holistic responses should have higher horizons and more objects in order to capture relationships between those objects, compared with analytic responses which should have fewer focal objects.

Fourth, a pronoun measure asked participants to think of a recent social occasion which they enjoyed, and to describe what happened in five sentences. Participants had five blank lines to write their description. Different examples before and after the game were given with equal numbers of independent (e.g. ‘I’) and interdependent (e.g. ‘we’) pronouns. Independent responses should have more independent pronouns relative to interdependent pronouns, while interdependent responses should have more interdependent pronouns relative to independent [25].

Finally, a questionnaire was taken from Singelis [35] which asks participants their agreement with statements indicative of independent or interdependent values on Likert scales. This questionnaire originally had 12 questions in each of the interdependent and independent categories. However, a re-analysis found that eight questions (four independent and four interdependent) clustered into a third group which can be interpreted as relating to hierarchy, with significant gender differences in this third group [36]. These questions were therefore omitted, leaving eight independent and eight interdependent questions. Four independent and four interdependent questions were given before the priming task, and the others afterwards.

#### 2.1.3. Materials: priming tasks

Both priming tasks were conducted at computer terminals within a small computer laboratory, and programmed using *z*-tree [37]. The collective priming task involved participants in groups of 3–5 playing the public goods game with punishment [38]. Parameters were adjusted for different group sizes (table 1). Participants were told that they were farmers who had enough seed to plant *E* acres of land per season. They had to decide whether to plant seeds in their private land, or to plant it in the communal land. The communal land has the benefit of shared effort and therefore yielded more crop per acre planted than did the private land, such that communal crops were multiplied by a factor of *b* (where *b* > 1). They could also punish each other for not planting enough seeds in the communal area, by devoting 0 to *P*_max_ of their crop units towards punishment. Each unit of their crop they put towards punishing another player was multiplied by *P*_mf_. Participants could see in each round how many units of seed other players had planted in the communal land, but they could not identify other players and were told the order in which contributions were presented changed between seasons. Players could see the history of their scores in a table on the right-hand side of the screen. The total number of crop units accrued at the end of the game was then converted into money using the conversion rate in table 1.

**Table 1.** Public goods game parameters.

		multiplication	maximum	punishment multiplication	conversion
group size, *N*	endowment, *E*	factor, *b*	punishment, *P*_max_	factor, *P*_mf_	rate (pence per unit)
3	20	1.5	10	3	1.5
4	30	2	15	3	0.8
5	20	2.5	10	3	1

The solitary priming task was a multi-armed bandit game. Participants were told they were fishermen and they had to catch fish every day to feed their family and sell on the market. There were four rivers: North, South, East and West, and only one river per day could be fished. The quality of the rivers changed: on any day, there was only one river that delivered large catches (between five and nine fish), while the other three delivered only between two and six fish. On average, a river would remain high yielding for 5 days in a row. After that the river would be depleted and another river would become the high yielding one. Every day the family needed to eat three fish, and the remaining number of fish would be sold at 1.25 pence per fish. This was the participants' reward.

#### 2.1.4. Participants

Eighty-four participants (68 female, 16 male) took part. Data from two participants were discarded because more than one dependent measure task was unfinished, leaving 41 participants in the cooperative condition and 41 in the solitary condition. Ages ranged from 17 to 38 years (mean = 19.58, s.d. = 2.829). Three to five participants took part in each session. All participants were students at Queen Mary University of London. Participants were paid between £4 and £10, depending on their score in the computer task. The study was approved by the Queen Mary Research Ethics Committee, reference QMREC2010/56.

#### 2.1.5. Procedure

Upon entering the laboratory, participants were allocated to a computer divided by a screen. Participants answered some background questions, gave their consent and filled out the five measures in the paper booklet for which they had 10 min. Then they read an introduction to the computer task which included questions to check their understanding of the game. After checking that everyone could produce the right answers, two practice rounds were played, followed by rounds in which money could be earned. After the game was finished, the second set of five measures was filled out in the paper booklet which also took 10 min. Then participants were debriefed, paid and were free to leave. Sessions did not exceed 1 h.

#### 2.1.6. Coding

In the categorization task, we coded the number of holistic choices out of all possible choices (analytic plus holistic). High values therefore indicate holistic responses, while low values indicate analytic responses. In the portrait choosing task, each face size was given a number from 1, largest face (most analytic), to 4, smallest face (most holistic). The dependent measure was the mean of choices in the five sets of pictures. Again, a high score indicates holistic cognition. The landscape drawing task gave two separate measures, horizon height and number of objects. For the horizon height, a line was drawn through the participant's horizon that approximately had as much surface area under it as above it. The dependent measure was the mean height of this line relative to the height of the entire drawing box. Higher values indicate more holistic responses. For the number of additional items, all extra items except the obligatory ones were counted separately, except for groups of very simply drawn items, such as flocks of V-shaped birds and grass dispersed throughout the drawing. Again, higher values indicate more holistic responses. In the pronoun task, we counted the number of independent and interdependent pronouns used. These were significantly negatively correlated both before and after the prime, so were combined into a single measure, the ratio of interdependent to independent pronouns. High values of this ratio indicate more interdependent relative to independent, and therefore more interdependence. Finally, self-construal gave two measures, one the mean of the independent values and one the mean of the interdependent values. These were not significantly correlated so were retained as separate measures. To reiterate, for all measures except independent self-construal, high values indicate more interdependent or holistic thinking.

#### 2.1.7. Analyses

Data were analysed by linear mixed-effects models fit by maximum likelihood accounting for random within-subject effects on repeated measures of the dependent variables and interaction effects between experimental activity condition (solitary versus collective) and time (pre-prime versus post-prime). A statistically significant activity × time interaction would support our hypothesis, and indicate that the measure changed following the prime in different directions depending on condition. Specifically, the ecocultural hypothesis predicts that the solitary condition should show a significant change towards independent/analytic responses following the prime, and the collective condition towards interdependent/holistic responses following the prime. Values for number of additional objects drawn were log transformed after adding one to eliminate zero values. Initial analyses, presented here, include only the hypothesized interaction between condition and the prime. Full analyses including model comparison and coefficients for best-fitting models (if any) including region of birth and gender as main and interaction effects are presented in electronic supplementary material, tables S1–S7. All analyses were performed using R statistical software v. 3.2.4 [39] and package nlme [40]. All data for Experiment 1 are included in electronic supplementary material, File S1, and all R scripts used for all analyses are included in electronic supplementary material, File S3.

### 2.2. Results and discussion

Contrary to the hypothesis, we found no interaction between time (pre-prime versus post-prime) and activity (solitary versus collective) for any of the dependent measures (table 2). For no measure was there a statistically significant shift towards greater independence following the solitary task, nor a shift towards greater interdependence following the collective task.

**Table 2.** Regression models for Experiment 1. High values indicate interdependence or holistic cognition for every measure except independent self-construal, where high values indicate independence. Reference category for time is pre-prime, for activity is collective. An effect of the activity (solitary versus collective) priming task in the manner predicted would be indicated by a significant time × activity interaction, such that values in the solitary condition shift towards independence and values in the collective condition shift towards interdependence.

	value	s.e.	*t*-value	*p*-value
*categorization*				
intercept	0.583	0.039	15.098	<0.001***
time: post-prime	−0.037	0.032	−1.141	0.257
activity: solitary	0.107	0.055	1.965	0.053
time × activity	−0.005	0.045	−0.108	0.915
*portrait selection*				
intercept	2.995	0.103	29.147	<0.001***
time: post-prime	−0.062	0.089	−0.697	0.488
activity: solitary	−0.061	0.145	−0.42	0.676
time × activity	0.163	0.126	1.295	0.199
*landscape: horizon height*				
intercept	0.572	0.028	20.085	<0.001***
time: post-prime	0.045	0.027	1.698	0.094
activity: solitary	0.085	0.04	2.128	0.037*
time × activity	−0.005	0.036	−0.137	0.891
*landscape: objects* (*logged*)				
intercept	1.728	0.122	14.12	<0.001***
time: post-prime	0.013	0.137	0.095	0.924
activity: solitary	−0.086	0.162	−0.531	0.597
time × activity	0.165	0.181	0.91	0.366
*pronouns*				
intercept	0.489	0.041	11.917	<0.001***
time: post-prime	0.02	0.05	0.404	0.687
activity: solitary	−0.005	0.058	−0.087	0.931
time × activity	−0.073	0.071	−1.031	0.306
*independent self-construal*				
intercept	0.32	0.022	14.452	<0.001***
time: post-prime	−0.014	0.025	−0.582	0.562
activity: solitary	−0.013	0.031	−0.419	0.676
time × activity	0.059	0.035	1.672	0.099
*interdependent self-construal*				
intercept	0.414	0.026	15.801	<0.001***
time: post-prime	−0.009	0.031	−0.3	0.765
activity: solitary	−0.067	0.037	−1.815	0.074
time × activity	0.018	0.044	0.394	0.694

Significance codes *p < 0.05, **p < 0.01, ***p < 0.001.

**Figure 1.**
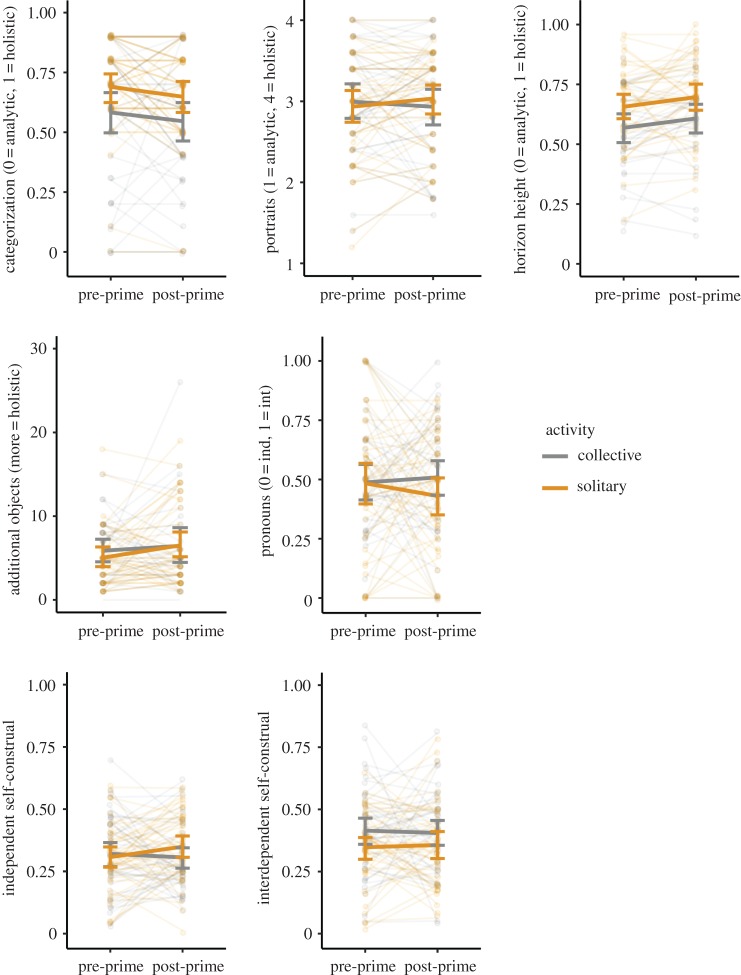
Changes in each of the seven dependent measures in response to the different activity conditions in Experiment 1. Each plot shows the shift from pre-prime to post-prime in the measure specified on the vertical axis (where int = interdependent, ind = independent), separately for participants primed with a solitary activity (orange) and participants primed with a collective activity (grey). Thick solid lines connect mean values pre- and post-prime, with error bars showing 95% CIs. Transparent lines and dots show each participant separately, with lines connecting each participant's pre- and post-prime response. Our hypothesis was that participants primed with solitary activity should become more analytic or independent, and participants primed with collective activity should become more holistic or interdependent. In the figure, this would be indicated by an upwards-slanting grey collective-prime line and a downwards-slanting orange solitary-prime line (except for independent self-construal which should have an upwards-slanting orange collective-prime line and a downwards-slanting grey solitary-prime line).

Figure 1 shows visually how the measures changed following the prime. Two measures, pronoun use and independent self-construal, showed a trend in the predicted direction, with participants primed with solitary activity shifting towards more independent responses, and participants primed with collective activity shifting towards more interdependent responses, although as just noted neither of these shifts were statistically significant. Categorization and horizon height showed slight (but statistically non-significant) pre-prime differences which were shifted post-prime, but there was no differential effect of the type of priming activity.

Note that this lack of statistical significance holds even without controlling for multiple tests. Given that seven models were run, one for each measure, on data from the same set of participants, we may decide to use a Bonferroni-corrected *α*-level of 0.05/7 = 0.007. Using this more conservative criterion fails to change our conclusion that the activity prime had no effect on any measure.

Some effects were statistically significant (at *α* = 0.05), but these do not relate directly to our hypothesis. Specifically, there was a statistically significant effect of activity for the landscape task horizon height measure (*p* = 0.037), and activity was approaching significance for categorization (*p* = 0.053), with higher horizons and more holistic categorization in the solitary condition. Note that these comparisons test the difference between activity conditions (solitary versus collective) at pre-prime, raising the possibility that despite random group assignment, there were systematic pre-prime differences in these measures. However, these effects were weak and would fail to reach statistical significance if using a Bonferroni-corrected *α* = 0.007. Moreover, our mixed models with time (pre-prime versus post-prime) as a repeated factor explicitly take into account both individual and group variation in the measures at pre-prime, which is to be expected in relatively small samples even with random group assignment.

To establish convergent and discriminant validity of subscales of our measures, we applied a multi-trait multi-method approach of scale validation from R package psy [41]. The results suggested the different dependent variables were measuring discriminant traits, with Pearson correlations below 0.3 between subscales (see electronic supplementary material, File S3). In addition, a principal components analysis of responses found six components with eigenvalues above 1.0 and nine with eigenvalues above 0.7 before the point of inflection on a scree plot (electronic supplementary material, File S3). This lack of correlation at the individual level between measures of culturally variable psychological processes is to be expected and is consistent with previous studies showing that group-level differences do not translate into individual-level differences [23].

Electronic supplementary material, tables S1–S7 and File S3 include full analyses of other predictors including participant gender, region of birth and performance in the task. The only robust effect found was an interaction between gender and the prime for the number of objects drawn in the landscape task, with men drawing significantly fewer objects after the prime compared with before (electronic supplementary material, table S1). This was the case for both activity conditions equally, however, and thus failed to relate to our hypothesis.

## 3. Experiment 2: origami folding tasks as primes

In Experiment 1, we tested the central prediction of the ecocultural hypothesis that working alone in a non-cooperative task versus working together in a cooperative task is the driving factor behind cultural variation in psychological processes relating to independence–interdependence and analytic–holistic cognition. In Experiment 1, however, participants were anonymous, and even in the collective condition sat at computer terminals and never interacted directly. In the real-life subsistence activities described and studied previously [21,22], people are not anonymous, and in collective situations will interact and communicate face-to-face in order to coordinate and cooperate. In Experiment 2, we therefore used face-to-face origami folding tasks, inspired by Insko *et al*. [28] but adapted for our purposes here. All participants made origami figures in exchange for points, which translated into monetary payoffs. Participants in the solitary condition could see others working alone on the same task. In the collective condition, payoffs were maximized if each group member contributed to sets of figures and if those contributions were coordinated in a certain way. This necessitated face-to-face communication and negotiation in order to coordinate each individuals' activity and effort in order to maximize payoffs.

Our main prediction was therefore that, as for Experiment 1, there should be a shift towards independent or analytic thinking following the solitary activity prime and towards interdependent or holistic thinking following the collective activity prime. However, we also took the opportunity to add a further manipulation, regarding payoffs and competitiveness. A recent study, using a similar natural contrast to Uskul *et al*. [21], found that fishing communities where most work was solitary were more competitive, as measured by a ball-throwing task, compared with neighbouring fishing communities where most work was collective [42]. Although that study described the solitarily working communities as ‘individualistic’ and the collectively working societies as ‘collectivistic’, no actual psychological measures were obtained. This suggests that activity and competitiveness may be confounded in comparisons such as Uskul *et al*. [21]. Communities where activity is solitary may be more psychologically independent because they are solitary, but alternatively (or additionally) because they are more competitive. Again, it is difficult to tease these confounds out using natural contrasts.

In Experiment 2, we therefore systematically manipulated competitiveness via the payoffs that participants received. Half of the solitary and half of the collective participants were rewarded based on absolute performance, where either individual performance (in the solitary condition) or group performance (in the collective condition) was translated directly into money using a formula converting the number of completed origami figures into pence. The other half of each condition was rewarded based on relative performance, where individuals (in the solitary condition) or groups (in the collective condition) were publicly ranked based on their performance, and given monetary rewards based on their rank. The latter relative payoff condition was predicted to increase competitiveness compared with the absolute payoff condition. If competitiveness is the driver of culturally variable psychological processes, we would expect relative payoffs to increase independent/analytic thinking, and absolute payoffs to increase interdependent/holistic thinking. It may also be that competitiveness and activity interact, with cognition only shifted towards independence when activity is solitary *and* payoffs are relative (i.e. competitiveness is high), and towards interdependence when activity is collective *and* payoffs are absolute (i.e. competitiveness is low). Our 2 × 2 × 2 design allows us to determine this via an interaction between activity and payoff.

### 3.1. Material and methods

#### 3.1.1. Design

Experiment 2 used a mixed 2 × 2 × 2 design. As before, priming was a within-participant factor with two levels (before versus after). There were two between-group factors each with two levels: activity (solitary versus collective) and payoffs (absolute versus relative). Participants were randomly assigned to one of the four between-participant conditions (solitary/absolute, solitary/relative, collective/absolute and collective/relative). The dependent variables were the same as for Experiment 1: answers on the same seven measures before and after playing the game. The standard ecocultural hypothesis predicts, as in Experiment 1, that participants in the collective condition would shift their answers towards greater interdependence, while participants in the solitary condition would shift towards greater independence.

An additional prediction, assuming that competition with others favours independence and coexistence with others favours interdependence, is that participants in the absolute condition would shift their answers towards greater interdependence, while participants in the relative condition would shift towards greater independence. If both processes work together, we would expect a shift towards independence only in the solitary/relative condition, and a shift towards interdependence only in the collective/absolute condition, with the effects cancelling out in the other conditions.

#### 3.1.2. Materials: priming tasks

Participants were seated in a university teaching laboratory. All participants in a session were assigned to the same condition (e.g. collective/absolute). Participants in the solitary condition were seated alone with as much space between them as possible. Participants in the collective condition were randomly assigned into groups of 3–5, where possible with people they did not know beforehand.

As in Experiment 1, all participants first took 10 min to complete the pre-task dependent measures separately. Dependent measures were identical to Experiment 1 except that for the categorization measure, participants were not asked to give a reason for their choice, as Experiment 1 revealed no cases in which reasons did not match our coding scheme. The origami session then began. During an initial training phase, each participant was given a square sheet of origami paper and printed instructions for how to make one of five origami figures: penguin, swan, fish, boat and piano. These instructions were taken from (now-defunct) websites and are shown in electronic supplementary material, File S6. They were all categorized as ‘Easy’ and estimated to take approximately 1 min to complete. Solitary participants in a session were all given the same figure, swan, to learn. In the collective condition, each member of a group was given a different figure to learn. The experimenter assisted any participants who needed help during the training phase.

Once every participant could produce a recognizable figure, the experiment began. The rest of the session was divided into three 4 min periods. During each 4 min period, participants made copies of their figures. Each participant could only make this assigned figure, and could not switch to another figure. Participants could choose one of three colours—red, blue or yellow—of origami paper with which to make their figure. This choice determined the rewards that were received (see below). At the end of every 4 min period, participants submitted the figures they had made to the experimenter in exchange for points. Figures could be submitted in sets. For solitary participants, sets contained three of the same figure, because solitary participants only learned to make one figure. For participants in groups, sets must contain one of each group member's learned figure. For example, for a group of three, there must be three different figures; for a group of four, there must be four different figures. This rule was imposed, so that each group member was forced to contribute equally to the rewarded sets, and to make the task a collective group activity.

The number of points earned by a set depended on the colour of the origami paper used to make them. The experimenter used rules unknown to the participants to give each set a score. These rules were: (i) two points for each red or blue figure in a set; (ii) one point for a yellow figure in a set; and (iii) a one-point bonus for having more red than blue figures in a set (with at least one of each). For example, a three-figure set of red, blue and yellow gave a score of five points (two points for each of the blue and red, plus one point for the yellow). A set of two red and one blue gave the maximum possible score for a set of three figures of seven points (two points for each of the red and blue figures, plus one point for having more red than blue). A set of three yellow gives the worst possible score for a set of three figures of three points (one point for each of the figures, and no bonus). The same rules were applied to sets of four or five produced by groups of four or five people. For example, a five-figure set of two reds, one blue and two yellows gave nine points (two points for each of the three reds and blues, one point for each of the two yellows and one bonus point for more reds than blues).

These rules were chosen to be sufficiently learnable as to give useful feedback—for example, it is relatively easy to work out that blues and reds are worth more than yellows—but not so transparent that the task is unengaging, given the bonus point which depends on the ratio of two colours. Crucially, the rules required the groups to coordinate their efforts and agree on the combination of colours to create as a set. Finally, for both solitary and collective participants, each figure produced that was not in a set gave ½ point. This was to ensure that participants kept working even when they or their group were unlikely to finish an entire set. Figures that the experimenter deemed unrecognizable were rejected, forfeiting the points from that set, in order to maintain a degree of focus and effort.

At the end of each 4 min period, the experimenter told each participant/group the number of points received for each set (but not each individual figure within a set) and recorded their total score. In the relative payoff conditions, the scores of each individual (in the solitary condition) or group (in the collective condition) were written on a whiteboard for public display to foster competition between individuals or groups. Each 4 min period typically resulted in two to four sets, depending on the speed with which figures were made. After each 4 min period, the experimenter cleared away all of the figures made during the previous period and began a new 4 min period. The experimenter recorded the total cumulative score over the three periods for each individual in the solitary condition and each group in the collective condition.

At the end of the origami session, participants had 10 min to complete the second post-task set of dependent measures, while their payment was calculated. Final cumulative scores were converted into money depending on the absolute versus relative payoff condition. In the absolute payoff conditions, the reward in pence was the total score multiplied by 12. In the collective absolute condition, this was divided equally among each group member. For example, a final score of 72 points obtained by a group of four gave a total reward of £8.64, or £2.16 per person (in addition to a £5 showing-up fee). In the relative payoff conditions, the individuals (in the solitary condition) or groups (in the collective condition) were ranked according to their final score and received a reward based on that rank. Individuals in sessions of three people received £5, £3 or £1, in sessions of four people received £5, £3.50, £2.50 or £1, and in sessions of five people received £5, £4, £3, £2 or £1. Each group member received the same amounts (e.g. every member of a group that was ranked second in a session of four groups received £3.50 each, in addition to the £5 showing-up fee).

Note that the collective condition is no longer technically a public goods game, as it was in Experiment 1. Each group member must contribute in order to form sets, which give much higher scores than figures that are not part of sets. One can free-ride by refusing to work (or work less) and getting the half-points from the other players' figures, but this temptation to defect is less than what one would get from working as hard as possible. It is, however, a cooperation/coordination game, in that everyone does better by contributing equally and maximally, and people in groups have to collectively discover and make the optimal combination of colours. This resembles the description of rice farming given by Talhelm *et al*. [22], where rice farming is distinguished from wheat farming mainly by the higher amount of effort that is required (more than that possible from a single family—hence the requirement in our origami game that everyone must contribute to get the high points associated with sets) and the need to coordinate planting schedules between families (hence the coordination aspect of our game, where group members must coordinate their colours).

#### 3.1.3. Participants

A total of 135 participants took part in the experiment (101 female, 33 male, 1 non-binary). Data from three participants were discarded as they withdrew from the study or did not complete the measures following the prime, leaving 30 in the solitary/absolute, 24 in the solitary/relative, 38 in the collective/absolute and 40 in the collective/relative conditions. Ages ranged from 18 to 32 years (mean = 20.6, s.d. = 2.4). Session sizes ranged from 1 (for solitary/absolute condition only) to 15 participants (median = 8, s.d. = 4.15). All participants were recruited through student employment and email networks at Durham University, UK, and were paid a showing-up fee of £5 plus the bonus for performance on the task (mean total payment = £8.23, s.d. = £1.82). Ethical approval was provided by the Durham University Department of Anthropology Ethics Committee.

#### 3.1.4. Analyses

Data were analysed by linear mixed-effects model fit by maximum likelihood accounting for random within-subject effects on repeated measures of the dependent variables and interaction effects between activity (solitary versus collective) and payoffs (absolute versus relative) conditions with the prime. As in Experiment 1, significant interactions between the prime and either condition would support our experimental hypothesis that the prime should differentially shift responses in the alternate conditions. We also tested for three-way interactions between activity (solitary versus collective), payoffs (relative versus absolute) and time (pre-prime versus post-prime), in case priming effects were only observed in certain combinations of conditions (e.g. only in solitary/relative and collective/absolute). None of these three-way interactions was significant, however, so we do not report them here, although they can be found in electronic supplementary material, tables S8–S14 and File S3.

Initial analyses, presented here, include only the hypothesized interaction between conditions and the prime. Further analyses, electronic supplementary material, tables S8–S14 and File S3, additionally included region of birth, *z*-transformed amount of payout, and gender as main and interaction effects. In no case did including these variables change the statistical significance of the prime × conditions interactions. Values for number of additional objects drawn were log transformed after adding one to eliminate zero values. All analyses were performed using R statistical software v. 3.2.4 [39]. All data for Experiment 2 are included in electronic supplementary material, File S2, and all R scripts used for all analyses are included in electronic supplementary material, File S3.

### 3.2. Results and discussion

**Figure 2.**
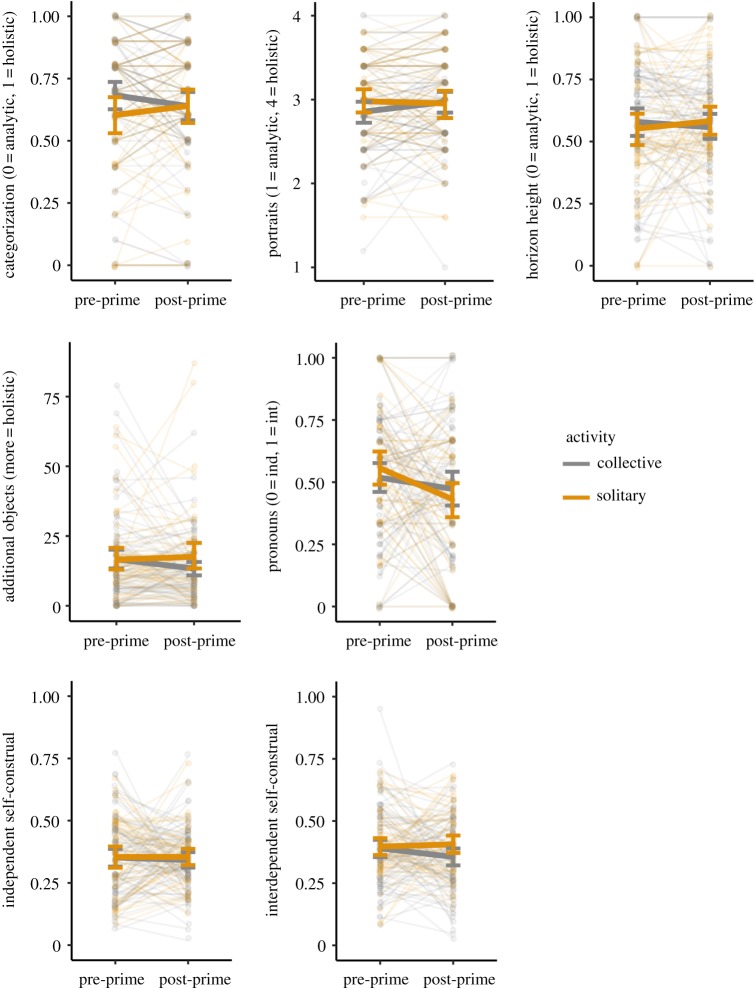
Changes in each of the seven dependent measures in response to the different activity conditions in Experiment 2. Each plot shows the shift from pre-prime to post-prime in the measure specified on the vertical axis (where int = interdependent, ind = independent), for participants primed with a solitary activity (orange) and participants primed with a collective activity (grey). Thick solid lines connect mean values pre- and post-prime, with error bars showing 95% CIs. Transparent lines and dots show each participant separately, with lines connecting each participant's pre- and post-prime response. Our hypothesis was that participants primed with solitary activity should become more analytic or independent, and participants primed with collective activity should become more holistic or interdependent. In the figure, this would be indicated by an upwards-slanting grey collective-prime line and a downwards-slanting orange solitary-prime line (except for independent self-construal which should have an upwards-slanting orange collective-prime line and a downwards-slanting grey solitary-prime line). See electronic supplementary material, figure S1 for visualization of the payoff condition from Experiment 2.

We focus first on our main hypothesis concerning the activity condition (solitary versus collective). Table 3 shows that only one of the seven measures, categorization, showed a statistically significant activity × time interaction, indicating that the activity prime differentially shifted performance on this task. However, as shown in figure 2, this was in the opposite direction to that predicted: participants showed more holistic categorization following priming by the solitary condition, and more analytic categorization following priming by the collective condition. This effect was also weak, and with a conservative Bonferroni-corrected *α* = 0.007 for seven tests would not be classed as statistically significant. Figure 2 also shows that for the other measures, there was little shift from pre- to post-prime: most of the solid lines are flat, except for pronoun use where both activity conditions shifted to become more independent. We again conclude, as in Experiment 1, that activity had no effect on thinking style.

**Table 3.** Regression models for Experiment 2. High values indicate interdependence or holistic cognition for every measure except independent self-construal, where high values indicate independence. Reference category for time is pre-prime, activity is collective and payment is absolute. An effect of activity (solitary versus collective) or payment (absolute versus relative) conditions on the measures would be indicated by a significant time × activity or time × payment interaction, respectively.

	value	s.e.	*t*-value	*p*-value
*categorization*				
intercept	0.677	0.038	17.805	<0.001***
payment: relative	0.012	0.045	0.263	0.793
time: post-prime	−0.047	0.029	−1.633	0.105
activity: solitary	−0.079	0.045	−1.738	0.085
time × payment	0.006	0.034	0.175	0.861
time × activity	0.079	0.035	2.275	0.025*
*portrait selection*				
intercept	1.021	0.042	24.261	<0.001***
payment: relative	0.012	0.036	0.332	0.740
time: post-prime	0.044	0.027	1.622	0.107
activity: solitary	0.026	0.036	0.722	0.472
time × payment	−0.004	0.033	−0.133	0.894
time × activity	−0.051	0.034	−1.521	0.131
*landscape: horizon height*				
intercept	0.536	0.04	13.518	<0.001***
payment: relative	0.081	0.041	1.964	0.052
time: post-prime	0	0.035	0.001	0.999
activity: solitary	−0.017	0.042	−0.405	0.686
time × payment	−0.042	0.042	−0.992	0.323
time × activity	0.058	0.043	1.357	0.177
*landscape: objects* (*logged*)				
intercept	2.141	0.148	14.475	<0.001***
payment: relative	0.518	0.177	2.92	0.004**
time: post-prime	0.152	0.145	1.045	0.298
activity: solitary	0.11	0.18	0.609	0.544
time × payment	−0.531	0.175	−3.024	0.003**
time × activity	0.157	0.178	0.882	0.380
*pronouns*				
intercept	0.505	0.039	13.058	<0.001***
payment: relative	0.04	0.047	0.862	0.390
time: post-prime	−0.019	0.047	−0.414	0.680
activity: solitary	0.033	0.047	0.7	0.485
time × payment	−0.057	0.056	−1.01	0.314
time × activity	−0.077	0.057	−1.344	0.181
*independent self-construal*				
intercept	0.341	0.021	16.558	<0.001***
payment: relative	0.016	0.025	0.633	0.528
time: post-prime	0.015	0.027	0.542	0.589
activity: solitary	0.006	0.025	0.257	0.798
time × payment	−0.041	0.033	−1.271	0.206
time × activity	0.004	0.033	0.131	0.896
*interdependent self-construal*				
intercept	0.406	0.022	18.82	<0.001***
payment: relative	−0.035	0.026	−1.36	0.175
time: post-prime	−0.054	0.029	−1.9	0.059
activity: solitary	0.007	0.026	0.28	0.780
time × payment	0.038	0.035	1.1	0.274
time × activity	0.046	0.035	1.31	0.194

Significance codes *p < 0.05, **p < 0.01, ***p < 0.001.

As expected, there were no pre-prime differences between activity conditions for any of the measures in Experiment 2, in accordance with our random group assignment. This is indicated by the ‘activity: solitary’ rows in table 3, none of which reached statistical significance even without using the Bonferroni correction.

Considering our second manipulation, absolute versus relative payoff, there was only one measure, objects drawn in the landscape task, which showed a statistically significant payoff × time interaction (table 3). This was in the predicted direction (electronic supplementary material, figure S1): participants in the absolute payoff condition drew more additional objects following the prime, and participants in the relative payoff condition drew fewer additional objects following the prime. This is in line with our prediction that absolute payment should favour holistic responses and relative payment favour analytic responses. Moreover, it would remain statistically significant even after applying a Bonferroni-corrected *α* = 0.007.

However, this may be because there was a large and unanticipated difference at pre-prime between absolute and relative groups in this measure, as indicated by the ‘payment: relative’ row in table 3, and as seen in electronic supplementary material, figure S1. We are unable to explain this initial large difference which occurred despite random assignment, but it may account for the significant interaction. From electronic supplementary material, figure S1 we can see that there were actually no differences between the groups at post-prime, suggesting simple regression to the mean.

Overall, the fact that only one of the seven measures showed a statistically significant effect of the payoff prime, and this sole significant interaction may be a side effect of unanticipated group differences for this specific measure at pre-prime and consequent regression to the mean, leads us to reject the ecocultural hypothesis when it is formulated in terms of payoff-induced competition.

As for Experiment 1, we explored convergent and discriminant validity of subscales of our measures using a multi-trait multi-method approach from R package psy [41]. As before, the results suggested the different dependent variables were measuring discriminant traits, with Pearson correlations again below 0.3 between subscales (electronic supplementary material, File S3). A principal components analysis found seven components with eigenvalues above 1.0 and nine with eigenvalues above 0.7 before the point of inflection on a scree plot (electronic supplementary material, File S3). This lack of individual-level correlation between measures that show cross-cultural variation is again to be expected [23].

As in Experiment 1, there were a few additional statistically significant effects related to gender, which we include in electronic supplementary material, tables S8–S14. Specifically, there were more independent pronouns used by men than by women overall, and more independent pronouns used after the prime by all participants compared with before the prime (electronic supplementary material, table S9). Men showed lower independent self-construal after the prime, but with no effect of either condition (electronic supplementary material, table S10). For interdependent self-construal, we found a three-way interaction between priming, gender and activity: men increased in interdependent self-construal under solitary conditions after the prime more than did women (electronic supplementary material, table S11). However, given the large number of tests run on our data in these subsequent analyses, and the lack of predicted effects for these measures, we are inclined to class these statistically significant effects as spurious.

## 4. General discussion

Our aim in this study was to use novel experimental priming methods to test the ecocultural hypothesis for the origin of cross-cultural variation in psychological processes. The ecocultural hypothesis [11,21,22] posits that independent modes of thinking (incorporating analytic cognition and individualistic self-construal) typical of modern-day Western societies has roots in the solitary herding of Ancient Greece, while the interdependent mode of thinking (incorporating holistic cognition and collectivistic self-construal) typical of modern-day East Asian societies has roots in the collective rice farming of Ancient China. We attempted to simulate these contrasting patterns of activity and interaction in the laboratory, to see whether we could shift thinking styles in the directions predicted by the ecocultural hypothesis: solitary activity was predicted to induce more independent or analytic thinking, while collective activity was predicted to induce more interdependent or holistic thinking. Experiment 1 used computer-based tasks from experimental economics, and Experiment 2 used origami folding tasks that were designed to elicit more direct and intense social interaction in the collective condition.

Across both experiments, just one of our seven measures shifted in the predicted direction following the priming tasks, and only in one of the experiments, failing overall to support the ecocultural hypothesis. The solitary tasks did not cause our participants' thinking style to become more independent or analytic, and the collective tasks did not cause participants' thinking style to become more interdependent or holistic, using psychological measures commonly shown to vary cross-culturally between Western and East Asian societies.

As with all negative findings, there are several possible explanations for why we failed to find the predicted effects. First, the ecocultural hypothesis may be incorrect. As we noted in the Introduction, the historical evidence is plausible but speculative [11], and contemporary cross-cultural comparisons [21,22] are few and not without issues of interpretation. It may be that some other historical difference gave rise to modern-day cross-cultural variation in thinking style. Alternative suggestions in the literature include different rates of climatic and socio-political change leading to differential reliance on social versus individual learning [43], different levels of pathogen exposure [44], location in a frontier region [20], the introduction of large-scale commerce [45] or the rise in socio-economic status [46]. Further historical and cross-cultural analyses, possibly combined with experimental tests as used here, will be needed to test between these alternative hypotheses.

Yet we would certainly not advocate rejecting the ecocultural hypothesis on the basis of this negative finding alone, without considering alternative reasons for our negative results. It is possible, for example, that our priming tasks were not appropriate or effective enough to constitute adequate tests of the ecocultural hypothesis. We made every effort to use priming tasks that we think capture the core elements of the ecocultural hypothesis: solitary working for one's own benefit versus collective working for a group. In Experiment 1, we used established computer-based tasks from experimental economics. In Experiment 2, we addressed the possibility that the collective activity needs to involve direct, face-to-face, non-anonymous interaction with others rather than simply working together. We therefore used face-to-face origami tasks which, based on our informal observations of the experimental sessions, did indeed induce intense communication, coordination and cooperation. In Experiment 2, we also manipulated whether payoffs were assigned in absolute terms or relative to other individuals/groups, with the latter designed to increase competitiveness. This was done in the light of recent findings that solitarily working communities are also more competitive than collectively working communities [42], suggesting that competitiveness and activity patterns may be confounded in previous natural contrasts. Yet still no effect of the prime was found, neither for activity alone, competitiveness alone, or both in combination.

Nevertheless, our priming tasks may still have lacked a crucial element of the ecocultural hypothesis. One possibility is extended joint attention. In Experiment 2, participants in the collective condition jointly decided the colour ratios of origami figures, but actually made their figures on their own. Perhaps if they had worked jointly on the same products, we would have fostered more interdependence. Another factor might be intensity of work. Rice farming was argued by Talhelm *et al*. [22] to be more work-intensive than wheat farming, which necessitates it being more collective, but perhaps it is work effort itself that is the crucial factor rather than collective action. This could be varied independently of activity pattern. Finally, our collective groups were mostly composed of strangers. Perhaps long-term social relationships are required to foster interdependence, and our experiments were too transient. Future studies might manipulate pre-existing participant familiarity as a factor. We offer our tasks for others to build on in future studies, and as a prompt to more precisely specify the necessary and sufficient components of the ecocultural hypothesis.

Another possibility is that the dependent measures that we used were not suitable for detecting the culturally variable dimensions of cognition and social orientation that we intended to capture. Given time constraints imposed by including our priming task, which had to be sufficiently lengthy to potentially prime our participants, we could not directly replicate the measures included in previous studies. Uskul *et al*.'s [21] measures, for example, took approximately 40 min. We also wanted to include as wide a range of measures as possible, including both non-self-report measures of analytic and holistic cognition and self-report-based questionnaire measures of social orientation, given evidence that both such measures vary cross-culturally but are not always perfectly correlated with each other [23]. Self-report questionnaire methods [35,36], while common, have been criticized for being unreliable [47]. We therefore used abbreviated measures of several tasks previously used in cross-cultural studies in order to maximize our chances of detecting an effect. However, future studies might focus on the specific measures used by Uskul *et al*. [21] and Talhelm *et al*. [22] to provide a more direct comparison to those naturalistic studies.

One might also take issue with our within-participant design, where we administered our dependent measures both before and after the priming task. This within-participant design was used to increase statistical power, given known individual variation in all of these measures [23]. Including time (pre-prime versus post-time) in our model as a repeated factor controlled for such individual variation. As a check, we confirmed that there were no systematic group differences at the pre-test stage (as indicated by the rows in tables 2 and 3 labelled ‘activity: solitary’), as expected given random group assignment. However, it is possible that familiarity with the measures generated carry-over effects to the post-prime measures, reducing the priming effect. We attempted to reduce this possibility by administering different parts of the measure before and after (e.g. one landscape was drawn before, and a different landscape was drawn after). But while participants could not simply recall their previous answers, it is possible that there was still some general carry-over. Future studies might administer the tasks once after the priming task, albeit with larger sample sizes to reduce the role of individual differences.

Even with a within-participant design, it is possible that our sample sizes were too small to detect the possible effect of activity. We note that our sample size of *n* = 84 in Experiment 1 and *n* = 135 in Experiment 2 is larger than 74% and 89%, respectively, of the 104 experiments included in a meta-analysis of independence–interdependence priming effects [24], making these two of the largest studies of their kind to date. Nevertheless, given the prevalence of under-powered studies in the literature, our sample size was probably too small to detect anything but moderate–large effects, and we recommend future studies use larger samples.

A final possibility is that the psychological constructs that we measured, and that cross-cultural psychologists typically measure in their cross-cultural comparisons, cannot be primed. While many previous experimental studies have shown independence–interdependence to be malleable and susceptible to priming [24], the recent ‘replication crisis’ in psychology [48], and particularly in social psychological studies using priming [49], casts doubt on this assumption. Before the recent crisis, it was implausible that the 104 experiments reported in 67 studies included in the meta-analysis in [24] could all be wrong; now it seems more plausible, given the extent of non-replication recently demonstrated [48]. We therefore recommend replications of previous studies that have claimed to have primed independence–interdependence in the laboratory, and offer our negative finding as a contribution to this re-evaluation.

It may be instead that the psychological processes in question are stable lifelong traits fixed early-on during development, such that they cannot be systematically shifted within the laboratory once people reach adulthood. Data from migrants support this assumption. First-generation migrants now living in Western countries who grew up in non-Western countries typically retain the psychological styles of their country of origin, while second-generation migrants (i.e. their children) are intermediate between their parents and the local Western thinking styles [9,10,50,51]. If psychological processes were susceptible to immediate priming, we would expect both first- and second-generation migrants living in Western societies to shift immediately to local values (unless migrants are entirely segregated within their minority group, which is perhaps tenable for some first-generation migrants, but extremely implausible for the second generation). If cross-culturally variable thinking styles are indeed developmentally fixed, or at least unmalleable over relatively short periods of time, then laboratory experiments cannot be used to test causal hypotheses for their origin.

Alternatively, it is possible that at least some previous priming effects are valid, but that priming only works by reminding people of contexts with which they are already familiar. In this study, we aimed to create the actual conditions hypothesized to have shifted cognition, i.e. people actually engaged in solitary versus collective activities. A standard priming task might instead have simply reminded participants of situations in their lives in which they have acted solitarily or collectively. While the latter might better resemble previous priming studies, we think that having participants actually engage in behavioural tasks represents a better test of the ecocultural hypothesis, given that this hypothesis explicitly states that solitary or collective *activity* fosters independence and interdependence, respectively. Even if memory-based priming had worked, it is unclear to us whether this would represent genuine support for the ecocultural hypothesis.

In conclusion, our study failed to support the ecocultural hypothesis for the origin of cross-cultural variation in psychological processes, by showing that activity patterns do not shift those psychological processes in the laboratory. While this may be interpreted as indicating that the ecocultural hypothesis is incorrect, it may also be because such processes are developmentally fixed and cannot be primed in the laboratory, contrary to the conclusions of a large literature in social psychology, or because our primes failed to correctly match the social conditions predicted by the ecocultural hypothesis. We hope that our negative finding is useful in guiding future experimental tests of the origin of culturally variable psychological characteristics, and in more precisely specifying the precise form of current hypotheses.

## Supplementary Material

Tables S1-S14

## Supplementary Material

Figure S1

## Supplementary Material

File S1

## Supplementary Material

File S2

## Supplementary Material

File S3

## Supplementary Material

File S4

## Supplementary Material

File S5

## Supplementary Material

File S6

## Data accessibility

All data are included as electronic supplementary material, Files S1 and S2. R scripts containing all statistical analyses are included as electronic supplementary material, File S3. Electronic supplementary material, File S4 contains definitions of variables in electronic supplementary material, Files S1 and S2. Electronic supplementary material, File S5 contains the dependent measures used. The data are also available from the Dryad Digital Repository (http://datadryad.org/resource/doi:10.5061/dryad.7j270) [52].
