# Supplementary material for: Experimental priming of independent and interdependent activity does not affect culturally variable psychological processes
Source: R Soc Open Sci. 2017 May 17;4(5):161025. doi: 10.1098/rsos.161025 (PMC5451795; doi:10.1098/rsos.161025)
Supplement: Tables S1-S14 [file rsos161025supp1.pdf]

Electronic Supplementary Material: Tables S1-S14

for

Magid, K., Sarkol, V. & Mesoudi, A.

Experimental priming of independent and interdependent activity does not affect culturally-variable psychological processes

Tables S1-S7: Model comparison and best-fitting model (if any) for the seven dependent measures in Experiment 1.

Tables S8-S14: Model comparison and best-fitting model (if any) for the seven dependent measures in Experiment 2.

Table S1: Experiment 1 Categorisation

a) ANOVA of linear mixed-effects models fit by maximum likelihood

Model		AIC	Test	L.Ratio	p-value	
1	Null model	-27.5			NA	
2	Priming	-28.5	1 vs 2	2.979	0.084	.
3	Solitary	-30.9	2 vs 3	4.448	0.035	*
4	Full model	-28.9	3 vs 4	0.012	0.913	

b) model coefficients: Solitary

Predictor		Value	Std.Error	t-value	p-value	
1	(Intercept)	0.584	0.037	15.877	<0.001	***
2	Response to prime	-0.039	0.023	-1.726	0.088	.
3	Solitary vs collective	0.105	0.05	2.118	0.037	*

Table S2: Experiment 1 Drawing of additional objects

a) ANOVA of linear mixed-effects models fit by maximum likelihood

Model		AIC	Test	L.Ratio	p-value	
1	Null model	266			NA	
2	Priming	267	1 vs 2	1.417	0.234	
3	Solitary	269	2 vs 3	0.001	0.977	
4	Sex	271	3 vs 4	0.228	0.633	
5	Priming x Solitary	272	4 vs 5	0.848	0.357	
6	Priming x Sex	270	5 vs 6	4.212	0.040	*
7	Solitary x Sex	271	6 vs 7	0.546	0.460	
8	Full model	266	7 vs 8	6.714	0.010	**

b) model coefficients: Priming x Sex

Predictor		Value	Std.Error	t-value	p-value	
1	(Intercept)	1.663	0.136	12.256	<0.001	***
2	Response to prime	0.231	0.144	1.609	0.113	
3	Solitary vs collective	-0.025	0.18	-0.137	0.891	
4	Male	0.312	0.298	1.048	0.299	
5	Response to prime x Solitary	-0.058	0.191	-0.304	0.762	
6	Response to prime x Male	-1.054	0.316	-3.339	0.001	**
7	Solitary x Male	-0.298	0.395	-0.754	0.454	
8	Response to prime x Solitary x Male	1.077	0.418	2.577	0.012	*

Table S3: Experiment 2 categorisation ratio interdependent/independent

a) ANOVA of linear mixed-effects models fit by maximum likelihood

Model		AIC	Test	L.Ratio	p-value	
1	Null model	-50.0			NA	
2	Priming	-48.4	1 vs 2	0.404	0.525	
3	Solitary	-47.4	2 vs 3	1.006	0.316	
4	Competitive	-45.5	3 vs 4	0.134	0.714	
5	Priming x Solitary	-48.7	4 vs 5	5.165	0.023	*
6	Priming x Competitive	-46.7	5 vs 6	0.031	0.860	
7	Solitary x Competitive	-46.2	6 vs 7	1.496	0.221	
8	Full model	-44.3	7 vs 8	0.056	0.813	

b) model coefficients: Priming x Solitary

Predictor		Value	Std.Error	t-value	p-value	
1	(Intercept)	0.675	0.037	18.326	<0.001	***
2	Response to prime	-0.044	0.022	-1.961	0.052	.
3	Solitary vs collective	-0.079	0.045	-1.737	0.085	.
4	Relative vs absolute	0.015	0.042	0.354	0.724	
5	Response to prime x Solitary vs collective	0.078	0.034	2.272	0.025	*

Table S4: Experiment 1 photo portrait size preferences

a) ANOVA of linear mixed-effects models fit by maximum likelihood

Model		AIC	Test	L.Ratio	p-value	
1	Null model	294			NA	
2	Priming	295	1 vs 2	0.096	0.757	
3	Solitary	297	2 vs 3	0.026	0.873	
4	Region of Birth	296	3 vs 4	9.164	0.057	.
5	Full model	297	4 vs 5	1.702	0.192	

b) model coefficients: Region of Birth

Predictor		Value	Std.Error	t-value	p-value	
1	(Intercept)	3.282	0.189	17.368	<0.001	***
2	Response to prime	0.02	0.064	0.303	0.762	
3	Solitary vs collective	0.02	0.129	0.159	0.874	
4	Other Regions of Birth vs UK	-0.079	0.251	-0.317	0.752	
5	Europe (non-UK) vs UK	-0.677	0.335	-2.021	0.047	*
6	South Asia vs UK	0.008	0.441	0.019	0.985	
7	South East Asia vs UK	-0.424	0.195	-2.174	0.033	*

Table S5: Experiment 1 pronoun ratio interdependent/independent ANOVA of linear mixed-effects models fit by maximum likelihood

Model		AIC	Test	L.Ratio	p-value
1	Null model	24.0			NA
2	Priming	25.8	1 vs 2	0.213	0.644
3	Solitary	26.9	2 vs 3	0.872	0.350
4	Sex	26.6	3 vs 4	2.348	0.125
5	Region of Birth	28.4	4 vs 5	6.211	0.184
6	Priming x Solitary	29.2	5 vs 6	1.107	0.293
7	Priming x Sex	30.6	6 vs 7	0.64	0.424
8	Solitary x Sex	32.1	7 vs 8	0.529	0.467
9	Full model	33.1	8 vs 9	0.987	0.320

Table S6: Experiment 1 Independent Self Construal ANOVA of linear mixed-effects models fit by maximum likelihood

Model		AIC	Test	L.Ratio	p-value	
1	Null model	-176		NA	NA	
2	Priming	-174	1 vs 2	0.676	0.411	
3	Solitary	-173	2 vs 3	0.395	0.530	
4	Sex	-173	3 vs 4	2.042	0.153	
5	Priming x Solitary	-174	4 vs 5	2.813	0.094	.
6	Priming x Sex	-173	5 vs 6	1.443	0.230	
7	Solitary x Sex	-171	6 vs 7	0.171	0.679	
8	Full model	-172	7 vs 8	2.245	0.134	

Table S7: Experiment 1 Interdependent Self Construal

a) ANOVA of linear mixed-effects models fit by maximum likelihood

Model		AIC	Test	L.Ratio	p-value	
1	Null model	-116		NA	NA	
2	Priming	-114	1 vs 2	0.000501	0.982	
3	Solitary	-116	2 vs 3	3.876062	0.049	*
4	Sex	-114	3 vs 4	0.081412	0.775	
5	Priming x Solitary	-112	4 vs 5	0.158694	0.690	
6	Priming x Sex	-112	5 vs 6	1.824116	0.177	
7	Solitary x Sex	-112	6 vs 7	2.265972	0.132	
8	Full model	-111	7 vs 8	0.592423	0.441	

b) model coefficients: Solitary

Predictor		Value	Std.Error	t-value	p-value	
1	(Intercept)	0.41	0.024	17.286	<0.001	***
2	Response to prime	-0.001	0.022	-0.031	0.975	
3	Solitary vs collective	-0.059	0.03	-1.972	0.052	.

Table S8: Experiment 1 Horizon height

a) ANOVA of linear mixed-effects models fit by maximum likelihood

Model		AIC	Test	L.Ratio	p-value	
1	Null model	-114			NA	
2	Priming	-118	1 vs 2	5.73	0.017	*
3	Solitary	-121	2 vs 3	5.252	0.022	*
4	Sex	-119	3 vs 4	0.116	0.734	
5	Region of Birth	-113	4 vs 5	1.731	0.785	
6	Priming x Solitary	-111	5 vs 6	0.029	0.865	
7	Priming x Sex	-109	6 vs 7	0.02	0.888	
8	Solitary x Sex	-108	7 vs 8	0.654	0.419	
9	Full model	-107	8 vs 9	0.907	0.341	

b) model coefficients: Solitary

Predictor		Value	Std.Error	t-value	p-value	
1	(Intercept)	0.573	0.027	21.43	<0.001	***
2	Response to prime	0.043	0.018	2.388	0.02	*
3	Solitary vs collective	0.082	0.036	2.312	0.024	*

Table S9: Experiment 2 photo portrait size preferences ANOVA of linear mixed-effects models fit by maximum likelihood

Model		AIC	Test	L.Ratio	p-value
1	Null model	-126			NA
2	Priming	-126	1 vs 2	1.629	0.202
3	Solitary	-124	2 vs 3	0	0.986
4	Relative	-122	3 vs 4	0.097	0.756
5	Priming x Solitary	-122	4 vs 5	2.331	0.127
6	Priming x Relative	-120	5 vs 6	0.018	0.893
7	Solitary x Relative	-118	6 vs 7	0.001	0.973
8	Full model	-116	7 vs 8	0.146	0.703

Table S10: Experiment 2 pronoun ratio interdependent/independent

a) ANOVA of linear mixed-effects models fit by maximum likelihood

Model		AIC	Test	L.Ratio	p-value	
1	Null model	56.8			NA	
2	Priming	51.1	1 vs 2	7.686	0.006	**
3	Solitary	53.1	2 vs 3	0.019	0.891	
4	Relative	54.9	3 vs 4	0.111	0.739	
5	Sex	52.3	4 vs 5	4.612	0.032	*
6	Full model	53.9	5 vs 6	0.4	0.527	

b) model coefficients: Sex

Predictor		Value	Std.Error	t-value	p-value	
1	(Intercept)	0.559	0.036	15.721	<0.001	***
2	Response to prime	-0.079	0.028	-2.772	0.006	**
3	Solitary vs collective	0	0.037	-0.012	0.99	
4	Relative vs absolute	0.003	0.037	0.07	0.944	
5	Male	-0.093	0.043	-2.145	0.034	*

Table S11: Experiment 2 Independent self-construal

a) ANOVA of linear mixed-effects models fit by maximum likelihood

**Model**		**AIC**	**Test**	**L.Ratio**	**p-value**	
1	Null model	912			NA	
2	Priming	914	1 vs 2	0.049	0.825	
3	Solitary	916	2 vs 3	0.22	0.639	
4	Relative	918	3 vs 4	0.061	0.805	
5	Sex	920	4 vs 5	0.007	0.932	
6	Priming x Solitary	922	5 vs 6	0.039	0.843	
7	Priming x Relative	922	6 vs 7	1.642	0.200	
8	Priming x Sex	918	7 vs 8	6.4	0.011	*
9	Solitary x Relative	918	8 vs 9	1.462	0.227	
10	Solitary x Sex	919	9 vs 10	1.049	0.306	
11	Competitve x Sex	920	10 vs 11	1.394	0.238	

b) model coefficients: Priming x Sex

**Predictor**		**Value**	**Std.Error**	**t-value**	**p-value**	
1	(Intercept)	3.119	0.209	14.945	<0.001	***
2	Response to prime	0.383	0.268	1.43	0.155	
3	Solitary vs collective	0.048	0.237	0.201	0.841	
4	Relative vs absolute	0.189	0.235	0.803	0.424	
5	Male	0.418	0.273	1.533	0.128	
6	Response to prime x Solitary	0.08	0.307	0.26	0.795	
7	Response to prime x Compeitive	-0.479	0.304	-1.575	0.118	
8	Response to prime x Male	-0.891	0.353	-2.524	0.013	*

Table S12: Experiment 2 Interdependent self-construal

a) ANOVA of linear mixed-effects models fit by maximum likelihood

Model		AIC	Test	L.Ratio	p-value	
1	Null model	943			NA	
2	Priming	944	1 vs 2	0.943	0.332	
3	Solitary	944	2 vs 3	2.379	0.123	
4	Relative	945	3 vs 4	0.749	0.387	
5	Sex	946	4 vs 5	1.111	0.292	
6	Priming x Solitary	946	5 vs 6	1.553	0.213	
7	Priming x Relative	947	6 vs 7	1.236	0.266	
8	Priming x Sex	945	7 vs 8	3.728	0.054	.
9	Solitary x Relative	947	8 vs 9	0	0.992	
10	Solitary x Sex	948	9 vs 10	1.064	0.302	
11	Competitve x Sex	950	10 vs 11	0.2	0.654	
12	Priming x Solitary x Relative	951	11 vs 12	0.561	0.454	
13	Priming x Solitary x Sex	946	12 vs 13	7.403	0.007	**
14	Priming x Relative x Sex	946	13 vs 14	2.196	0.138	
15	Full model	949	14 vs 15	0.977	0.613	

b) model coefficients: Priming x Solitary x Sex

Predictor		Value	Std.Error	t-value	p-value	
1	(Intercept)	3.524	0.252	13.993	<0.001	***
2	Response to prime	-0.013	0.318	-0.039	0.969	
3	Solitary vs collective	0.621	0.391	1.589	0.115	
4	Relative vs absolute	-0.215	0.337	-0.639	0.524	
5	Male	1.094	0.443	2.469	0.015	*
6	Response to prime x Solitary	-0.403	0.504	-0.798	0.426	
7	Response to prime x Compeitive	0.075	0.415	0.18	0.858	
8	Response to prime x Male	-1.572	0.496	-3.169	0.002	**
9	Solitary x Relative	-0.427	0.504	-0.846	0.399	
10	Solitary x Male	-1.418	0.586	-2.422	0.017	*
11	Competitve x Male	0.18	0.454	0.397	0.692	
12	Response to prime x Solitary x Relative	0.743	0.656	1.133	0.259	
13	Response to prime x Solitary x Male	2.029	0.753	2.696	0.008	**

Table S13: Experiment 2 Horizon height

a) ANOVA of linear mixed-effects models fit by maximum likelihood

Model		AIC	Test	L.Ratio	p-value	
1	Null model	-35.7			NA	
2	Priming	-33.7	1 vs 2	0.027	0.869	
3	Solitary	-31.7	2 vs 3	0.006	0.937	
4	Relative	-32.3	3 vs 4	2.614	0.106	
5	Payout	-30.3	4 vs 5	0.013	0.911	
6	Region of Birth	-33.4	5 vs 6	11.093	0.026	*
7	Priming x Solitary	-33.4	6 vs 7	2.008	0.156	
8	Priming x Relative	-32.5	7 vs 8	1.015	0.314	
9	Priming x Payout	-31.1	8 vs 9	0.639	0.424	
10	Solitary x Relative	-29.1	9 vs 10	0.019	0.891	
11	Solitary x Payout	-30.2	10 vs 11	3.075	0.079	.
12	Competitve x Payout	-28.4	11 vs 12	0.186	0.667	
13	Priming x Solitary x Relative	-26.5	12 vs 13	0.093	0.760	
14	Priming x Solitary x Payout	-24.5	13 vs 14	0.025	0.873	
15	Priming x Relative x Payout	-22.6	14 vs 15	0.089	0.765	
16	Full model	-24.6	15 vs 16	6.015	0.049	*

b) model coefficients: Region of Birth

Predictor		Value	Std.Error	t-value	p-value	
1	(Intercept)	0.5	0.057	8.737	<0.001	***
2	Response to prime	0.004	0.021	0.169	0.866	
3	Solitary vs collective	0.012	0.036	0.345	0.731	
4	Relative vs absolute	0.068	0.035	1.931	0.056	.
5	Payout amount (z-transformed)	-0.005	0.018	-0.264	0.792	
6	Other Regions of Birth vs UK	-0.043	0.089	-0.48	0.664	
7	Europe (non-UK) vs UK	0.223	0.086	2.596	0.081	.
8	South Asia vs UK	-0.017	0.058	-0.282	0.796	
9	South East Asia vs UK	0.049	0.055	0.886	0.441	

c) model coefficients: Full model

Predictor		Value	Std.Error	t-value	p-value	
1	(Intercept)	0.496	0.064	7.764	<0.001	***
2	Response to prime	0.018	0.045	0.408	0.684	
3	Solitary vs collective	-0.002	0.062	-0.038	0.97	
4	Relative vs absolute	0.101	0.056	1.79	0.076	.
5	Payout amount (z-transformed)	-0.052	0.065	-0.802	0.424	
6	Other Regions of Birth vs UK	-0.061	0.09	-0.674	0.549	
7	Europe (non-UK) vs UK	0.232	0.087	2.672	0.076	.
8	South Asia vs UK	-0.02	0.058	-0.343	0.755	
9	South East Asia vs UK	0.041	0.056	0.735	0.516	

c) model coefficients: Full model

Predictor		Value	Std.Error	t-value	p-value	
10	Response to prime x Solitary	0.046	0.065	0.706	0.482	
11	Response to prime x Relative	-0.053	0.059	-0.895	0.373	
12	Response to prime x Payout	0.087	0.068	1.277	0.204	
13	Solitary x Relative	-0.022	0.085	-0.259	0.796	
14	Solitary x Payout	0.037	0.071	0.528	0.599	
15	Relative x Payout	0.138	0.081	1.695	0.093	.
16	Response to prime x Solitary x Relative	-0.003	0.09	-0.034	0.973	
17	Response to prime x Solitary x Payout	-0.078	0.074	-1.057	0.292	
18	Response to prime x Relative x Payout	-0.12	0.086	-1.392	0.167	
19	Pre-prime x Solitary x Relative x Payout	-0.243	0.104	-2.336	0.021	*
20	Response to prime x Solitary x Relative x Payout	-0.072	0.106	-0.685	0.494	

Table S14: Experiment 2 Additional objects drawn

a) ANOVA of linear mixed-effects models fit by maximum likelihood

Model		AIC	Test	L.Ratio	p-value	
1	Null model	746			NA	
2	Priming	748	1 vs 2	0.227	0.634	
3	Solitary	749	2 vs 3	1.002	0.317	
4	Relative	748	3 vs 4	2.579	0.108	
5	Payout	750	4 vs 5	0.393	0.531	
6	Region of Birth	751	5 vs 6	6.956	0.138	
7	Priming x Solitary	752	6 vs 7	1.044	0.307	
8	Priming x Relative	745	7 vs 8	9.155	0.002	**
9	Priming x Payout	747	8 vs 9	0.163	0.687	
10	Solitary x Relative	749	9 vs 10	0	0.997	
11	Solitary x Payout	745	10 vs 11	5.405	0.020	*
12	Relative x Payout	747	11 vs 12	0.301	0.583	
13	Priming x Solitary x Relative	749	12 vs 13	0.053	0.817	
14	Priming x Solitary x Payout	750	13 vs 14	0.621	0.431	
15	Priming x Relative x Payout	752	14 vs 15	0.28	0.597	
16	Full model	751	15 vs 16	5.054	0.080	.

b) model coefficients: Priming x Relative

Predictor		Value	Std.Error	t-value	p-value	
1	(Intercept)	2.32	0.261	8.886	<0.001	***
2	Response to prime	0.152	0.146	1.04	0.301	
3	Solitary vs collective	0.082	0.183	0.445	0.657	
4	Relative vs absolute	0.497	0.179	2.768	0.007	**
5	Payout amount (z-transformed)	0.063	0.08	0.794	0.429	
6	Other Regions of Birth vs UK	0.529	0.399	1.326	0.277	
7	Europe (non-UK) vs UK	0.053	0.384	0.139	0.898	
8	South Asia vs UK	-0.156	0.261	-0.598	0.592	
9	South East Asia vs UK	-0.305	0.247	-1.238	0.304	
10	Priming x Solitary	0.156	0.18	0.865	0.388	
11	Priming x Relative	-0.533	0.177	-3.01	0.003	**

c) model coefficients: Solitary x Payout

Predictor		Value	Std.Error	t-value	p-value	
1	(Intercept)	2.401	0.272	8.819	<0.001	***
2	Response to prime	0.157	0.148	1.065	0.289	
3	Solitary vs collective	0.064	0.242	0.266	0.791	
4	Relative vs absolute	0.422	0.223	1.896	0.06	.
5	Payout amount (z-transformed)	0.325	0.153	2.124	0.036	
6	Other Regions of Birth vs UK	0.541	0.394	1.374	0.263	

c) model coefficients: Solitary x Payout

Predictor		Value	Std.Error	t-value	p-value	
7	Europe (non-UK) vs UK	0.079	0.381	0.208	0.848	
8	South Asia vs UK	-0.169	0.258	-0.654	0.56	
9	South East Asia vs UK	-0.303	0.244	-1.245	0.302	
10	Response to prime x Solitary	0.14	0.184	0.76	0.449	
11	Response to prime x Relative	-0.531	0.178	-2.983	0.003	**
12	Response to prime x Payout	0.038	0.091	0.42	0.675	
13	Solitary x Relative	0.008	0.315	0.024	0.981	
15	Solitary x Payout	-0.396	0.173	-2.284	0.024	*
