# Supplementary figures and images for: Experimental priming of independent and interdependent activity does not affect culturally variable psychological processes
Source: R Soc Open Sci. 2017 May 17;4(5):161025. doi: 10.1098/rsos.161025 (PMC5451795; doi:10.1098/rsos.161025)

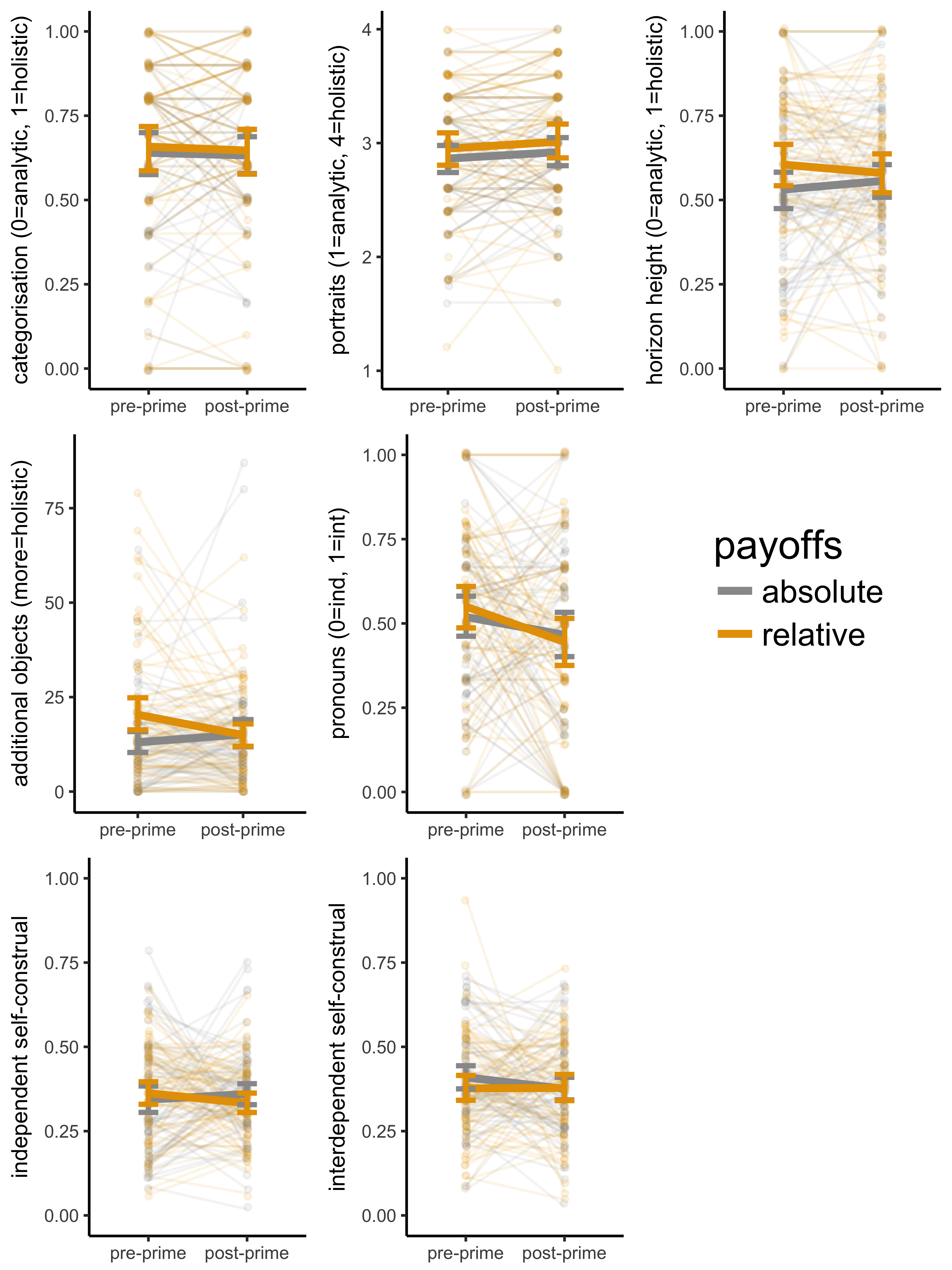

Supplement: Figure S1 [file rsos161025supp2.png]
