# Supplementary material for: Experimental priming of independent and interdependent activity does not affect culturally variable psychological processes
Source: R Soc Open Sci. 2017 May 17;4(5):161025. doi: 10.1098/rsos.161025 (PMC5451795; doi:10.1098/rsos.161025)
Supplement: File S4 [file rsos161025supp6.pdf]

Definitions of variable names in the dataset for Magid, Sarkol & Mesoudi 'Experimental priming of independent and interdependent activity does not affect culturally-variable psychological processes'

A. Experiment 1 dataset: file MagidSarkolMesoudi_ESM_FileS1_exp1data.csv

Variable name	Definition
participant	Participant identification number
date	Date of experiment
session	Session number
activity	Experimental condition: solitary or collective
n	Number of participants in session
meancontribution	Mean contribution in allotted units to the Public Goods Game (collective condition only)
meanpunishgiven	Mean number of units allocated to punishment in the Public Goods Game (PGG)
meanpunishreceived	Mean number of units deducted due to punishment by others in the PGG
pggearnings	Amount of money earned in the PGG
pggearnrank	Ranking of all participants according to pggearnings
pggearnrelativerank	The variable pggearnrank scaled from 0-1
pggpunishmentgivenrank	Ranking of all participants according to meanpunishgiven
pggpunishmentgivenrelativerank	The variable pggpunishmentgivenrank scaled from 0-1
pggpunishreceivedrank	Ranking of participants according to meanpunishreceived
pggpunishreceivedrelativerank	The variable pggpunishreceivedrank scaled from 0-1
rightchoice	Mean number of correct choices in the Multi-Armed Bandit (MAB) game (solitary condition only)
rightchoicerank	Ranking according to number of correct choices
rightchoicerelativerank	The variable rightchoicerank scaled from 0-1
sortgoodnessofparticipant	Mean success in either the PGG or MAB game, scaled 0-1
goodnessofparticipant	Ranking according to sortgoodnessofparticipant
beholcategorisation	Before the prime, number of holistic choices in the categorisation task
afholcategorisation	After the prime, number of holistic choices in the categorisation task
bepictures	Before the prime, mean number of holistic portrait choices

afpictures	After the prime, mean number of holistic portrait choices
besingpronouns	Before the prime, number of independent pronouns generated
afsingpronouns	After the prime, number of independent pronouns generated
beplurpronouns	Before the prime, number of interdependent pronouns generated
afplurpronouns	After the prime, number of interdependent pronouns generated
beplursingpronouns	Ratio of besingpronouns and beplurpronouns before the prime
afplursingpronouns	Ratio of besingpronouns and beplurpronouns after the prime
behorizon	Before the prime, horizon height in the landscape drawing task scaled 0-1
afhorizon	After the prime, horizon height in the landscape drawing task scaled 0-1
beadditems	Before the prime, the number of additional items in the landscape drawing task
afadditems	After the prime, the number of additional items in the landscape drawing task
beindvalues	Mean score on the independent / individualism items of the questionnaire before the prime
afindvalues	Mean score on the independent / individualism items of the questionnaire after the prime
becolvalues	Mean score on the interdependent / collectivism items of the questionnaire before the prime
afcolvalues	Mean score on the interdependent / collectivism items of the questionnaire after the prime
age	Age of participant
sex	Gender of the participant
study	Study subject of the student participant
year	Study year of the undergraduate participants, or years in university education for postgraduates
familiaritywithgroup	Whether participants were familiar with their fellow group members before the experiment
country.born	Country in which participant was born
moveukage	If not born in the UK, age at which participant moved to the UK
parent1m.country.born	Father's country of birth
parent2f.country.born	Mother's country of birth
grandparentsborn1 - grandparentsborn4	Country in which grandparents were born
region.born	Region in which participant was born: UK/Ire, Europe (non-UK), SE Asia, Africa, N Africa / Mid-east, Russia/C Asia, S America/Carribean
pt.1_categ	Proportionate variable of pre-prime categorical responses (0=no ind, 1=all ind, .5=evenly ind and int.)
pt.2_categ	Proportionate variable of post-prime categorical responses (0=no ind, 1=all ind, .5=evenly ind and int.)

pt.1_picsize	Pre-prime mean photo portrait face choice size
pt.2_picsize	Post-prime mean photo portrait face choice size
pron.ind_pt.1	Pre-prime summed number of singular "ind" pronouns used
pron.ind_pt.2	Post-prime summed number of singular "ind" pronouns used
pron.int_pt.1	Pre-prime summed number of plural "int" pronouns used
pron.int_pt.2	Post-prime summed number of plural "int" pronouns used
rat_pron_pt.1	Pre-prime ratio of singular "ind"/total number of pronouns used
rat_pron_pt.2	Post-prime ratio of singular "ind"/total number of pronouns used
selfcon.ind_time.1	Pre-prime mean independent self-construal response, 0="strongly agree"; 9.5="strongly disagree"
selfcon.ind_time.2	Post-prime mean independent self-construal response, 0="strongly agree"; 9.5="strongly disagree"
selfcon.int_time.1	Pre-prime mean interdependent self-construal response, 0=most interdependent "strongly agree"; 9.5=least interdependent"strongly disagree"
selfcon.int_time.2	Post-prime mean interdependent self-construal response, 0=most interdependent "strongly agree"; 9.5=least interdependent"strongly disagree"
hor_time.1	Pre-prime ratio of horizon height/box height
hor_time.2	Post-prime ratio of horizon height/box height
addo_time.1	Pre-prime total number of additional objects
addo_time.2	Post-prime total number of additional objects
payout	Payout in pounds to participant
region_born.red	Reduced region born categories: "UK/Ire", "Other", "Europe (non-UK)", "S Asia", "SE Asia"

B. Experiment 2 dataset: file MagidSarkolMesoudi_ESM_FileS2_exp2data.csv

Variable name	Definition
date	Date of experiment
session	Session number
condition	Experimental condition: A (solitary absolute); B (solitary relative); C (collective absolute); D (group relative)
n	Number of participants in session
participant	Team or participant designation
score_r1-score_r3	Number of points earned for task performance each round
score_total	Total score in points earned for task performance
payout	Total amount paid to participant in £GBP, (£5.00 honorarium + performance bonus)
rank	Relative conditions only, rank out of all participants (solitary) or teams (collective)
version	Questionniare version used
pt.1_categ_q1-<br>pt.1_categ_q10;<br>pt.2_categ_q1-<br>pt.2_categ_q10	Categorical response: Analytical rule="Ind", Holistic rule="int"
pt.1_categ_r1-<br>pt.1_categ_r10;<br>pt.2_categ_r1-<br>pt.2_categ_r10;	Reason given (if any) for choice of categorisation response
pt.1_sec2_pic1-<br>pt.1_sec2_pic5;<br>pt.2_sec2_pic1-<br>pt.2_sec2_pic5	Photo portrait choice response of 1-4 photos
pt.1_sec3_s1_sing-<br>pt.1_sec3_s5_sing;<br>pt.2_sec3_s1_sing-<br>pt.2_sec3_s5_sing	Number of singular (independent) pronouns used per sentence 1-5
pt.1_sec3_s1_plur-<br>pt.1_sec3_s5_plur;<br>pt.2_sec3_s1_plur-<br>pt.2_sec3_s5_plur	Number of plural (interdependent) pronouns used per sentence 1-5

pt.1_sec4_q1-<br>pt.1_sec4_q8;<br>pt.2_sec4_q1-<br>pt.2_sec4_q8;	Distance (cm) of participant's mark on a line from left "strongly agree"=0.0 to right "strongly disagree"=9.5
pt.1_sec5_horizon_height	Height (cm) of average horizon meeting point on left and right margins, from bottom of the drawing
pt.1_sec5__boxheight	Total height (cm) of box for drawing task
pt.1_sec5__additional_items	total count of objects in drawing added outside of requested five objects
age	Age in years
sex	Sex "Male" or "Female"
country.born	Response to "In which country were you born?"
parent1m.country.born	Response to "In which country (or countries) were your parents born?" Mother:
parent2f.country.born	Response to "In which country (or countries) were your parents born?" Father:
other.countries1-<br>other.countries3;<br>age_moved1-age_moved3	Response to "If you have lived in more than one country in your lifetime, please list those coutnries and give the age you were when you moved to each country" (Free response to fill as many as required)
occupation	Response to "What is your current occuption (If you are retired, please give your most recent occupation at the time of retirement)"
mother_occupation	Response to "What are your parents' current or (if retired) most recent occuptions?" Mother:
father_occupation	Response to "What are your parents' current or (if retired) most recent occuptions?" Father:
participant	Participant id number
activity	Solitary vs. collective condition
payoffs	Relative vs. absolute payout condition
gender	Including case of "non-binary"
region_born	Participant country born categorised by regions: "UK/Ire", "Africa", "Europe (non-UK)", "N Africa/Mid-East", "N America", "Russia/C Asia", "S America/Caribbean", "S Asia", "SE Asia"
mother_region_born	Participant's mother's country born categorised by regions: "UK/Ire", "Africa", "Europe (non-UK)", "N Africa/Mid-East", "N America", "Russia/C Asia", "S America/Caribbean", "S Asia", "SE Asia"
father_region_born	Participant's father's country born categorised by regions: "UK/Ire", "Africa", "Europe (non-UK)", "N Africa/Mid-East", "N America", "Russia/C Asia", "S America/Caribbean", "S Asia", "SE Asia"
pt.1_int_categorisation	Pre-prime summed number of categorical responses: Holistic rule="int"
pt.1_ind_categorisation	Pre-prime summed number of categorical responses: Analytic rule="ind"
pt.2_int_categorisation	Post-prime summed number of categorical responses: Holistic rule="int"

pt.2_ind_categorisation	Post-prime summed number of categorical responses: Analytic rule="ind"
chg_categ.int	Change in number of holistic "int" responses following prime
chg_categ.ind	Change in number of analytic "ind" responses following prime
pt.1_categ	Proportionate variable of pre-prime categorical responses (0=no ind, 1=all ind, .5=evenly ind and int.); calculation: pt.1_ind_categorisation / (pt.1_ind_categorisation + pt.1_int_categorisation)
pt.2_categ	Proportionate variable of post-prime categorical responses (0=no ind, 1=all ind, .5=evenly ind and int.); calculation: pt.2_ind_categorisation / (pt.2_ind_categorisation + pt.2_int_categorisation)
chg_categ	Change in proportion of categorical ind/int responses following prime
pt.1_picnum1	Numeric variables of photo portrait face choice size on 4 point scale with 1=smallest face (highest int) to 4=largest (highest ind)
pt.1_picsize	Pre-prime mean photo portrait face choice size
pt.2_picsize	Post-prime mean photo portrait face choice size
chg_pic	Change in mean photo portrait face choice size following prime
pron.ind_pt.1	Pre-prime summed number of singular "ind" pronouns used
pron.ind_pt.2	Post-prime summed number of singular "ind" pronouns used
pron.int_pt.1	Pre-prime summed number of plural "int" pronouns used
pron.int_pt.2	Post-prime summed number of plural "int" pronouns used
chg_pron.ind	Change in total number of singular "ind" pronouns used following prime
chg_pron.int	Change in total number of plural "int" pronouns used following prime
rat_pron_pt.1	Pre-prime ratio of singular "ind"/total number of pronouns used
rat_pron_pt.2	Post-prime ratio of singular "ind"/total number of pronouns used
chg_rat_pron	Change in ratio of singular "ind" to total pronouns used following prime
selfcon.ind_time.1	Pre-prime mean independent self-construal response, 0="strongly agree"; 9.5="strongly disagree"
selfcon.ind_time.2	Post-prime mean independent self-construal response, 0="strongly agree"; 9.5="strongly disagree"
selfcon.int_time.1	Pre-prime mean interdependent self-construal response, 0=most interdependent "strongly agree"; 9.5=least interdependent"strongly disagree"
selfcon.int_time.2	Post-prime mean interdependent self-construal response, 0=most interdependent "strongly agree"; 9.5=least interdependent"strongly disagree"
chg_selfcon.ind	Change in mean independent self-construal rating following prime
chg_selfcon.int	Change in mean interdependent self-construal rating following prime
rat_selfcon.ind_time.1	Pre-prime ratio of independent/(interdependent + independent) self construal ratings

rat_selfcon.ind_time.2	Post-prime ratio of independent/(interdependent + independent) self construal ratings
chg_rat_selfcon.ind	Change in ratio of independent/(interdependent + independent) self construal ratings following prime
selfcon.ver_time.1	Pre-prime mean vertical self-construal response, 0="strongly agree"; 9.5="strongly disagree"
selfcon.ver_time.2	Post-prime mean vertical self-construal response, 0="strongly agree"; 9.5="strongly disagree"
selfcon.hor_time.1	Pre-prime mean horizontal self-construal response, 0=most horizontal "strongly agree"; 9.5=least horizontal "strongly disagree"
selfcon.hor_time.2	Post-prime mean horizontal self-construal response, 0=most horizontal "strongly agree"; 9.5=least horizontal "strongly disagree"
chg_selfcon.ver	Change in mean vertical self-construal rating following prime
chg_selfcon.hor	Change in mean horizontal self-construal rating following prime
rat_selfcon.hor_time.1	Pre-prime ratio of horizontal/(vertical + horizontal) self construal ratings
rat_selfcon.hor_time.2	Post-prime ratio of horizontal/(vertical + horizontal) self construal ratings
chg_rat_selfcon.hor	Change in ratio of horizontal/(vertical + horizontal) self construal ratings following prime
hor_time.1	Pre-prime ratio of horizon height/box height
hor_time.2	Post-prime ratio of horizon height/box height
chg_hor	Change in ratio of horizon height/box height following prime
addo_time.1	Pre-prime total number of additional objects
addo_time.2	Post-prime total number of additional objects
chg_addo	Change in number of additional objects following the prime
region_born.red	Reduced region born categories: "UK/Ire", "Other", "Europe (non-UK)", "S Asia", "SE Asia"
