# Supplementary material for: Experimental priming of independent and interdependent activity does not affect culturally variable psychological processes
Source: R Soc Open Sci. 2017 May 17;4(5):161025. doi: 10.1098/rsos.161025 (PMC5451795; doi:10.1098/rsos.161025)
Supplement: File S5 [file rsos161025supp7.pdf]

Thank-you for agreeing to take part in this study.

There are five tasks, followed by a few simple questions about yourself.

Remember that it is not a test, and there are no right or wrong answers to any of the questions.

Please complete it in your own time, but try not to take too long on any one section.

Part 1

1. Which of the words belongs most to the first word? Please circle your choice.

	Choose between...	
**Dog**	Bone	Cat
**Spider**	Web	Grasshopper
**Police car**	Van	Policeman
**Tennis shoe**	Foot	Boot
**Labrador**	Poodle	Dog food
**Birthday cake**	Present	Muffin
**Cow**	Pig	Milk
**Ring**	Necklace	Hand
**Cup**	Kettle	Glass
**Train**	Bus	Tracks

2. Please indicate which of each four pictures you like best.

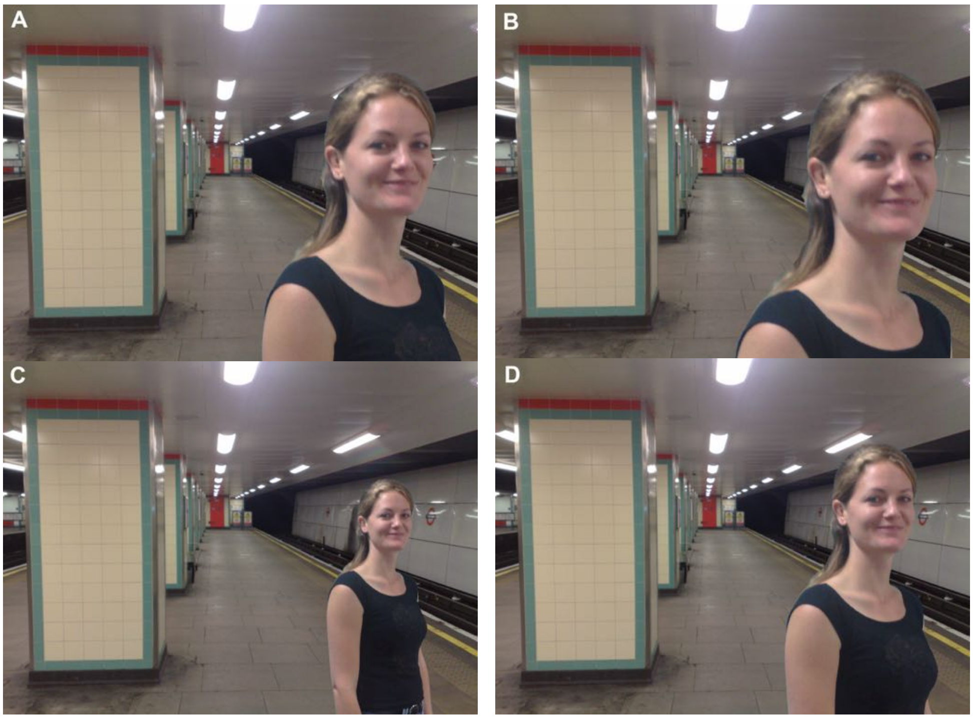

Which picture do you prefer? A/B/C/D

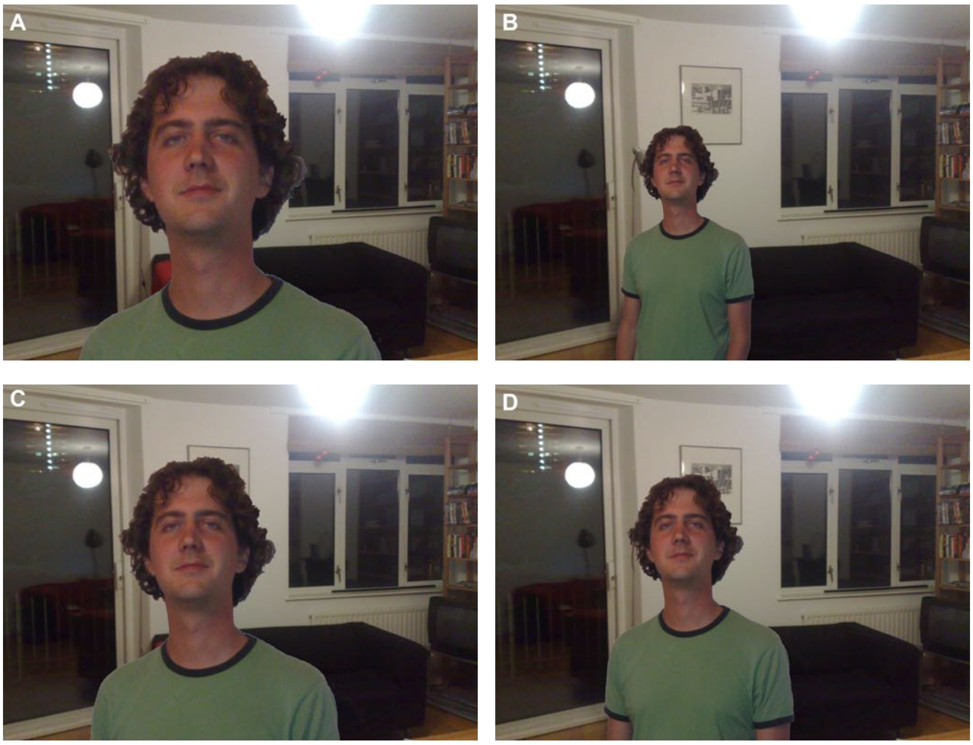

Tasks v.1: Paper Folding

Which picture do you prefer? A/B/C/D

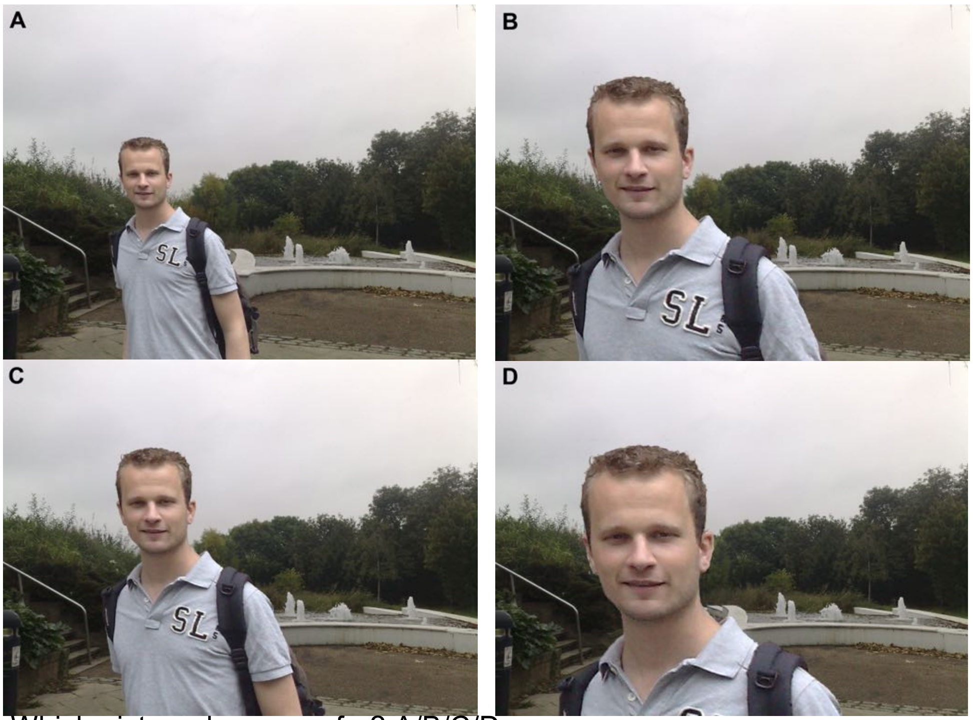

Which picture do you prefer? A/B/C/D

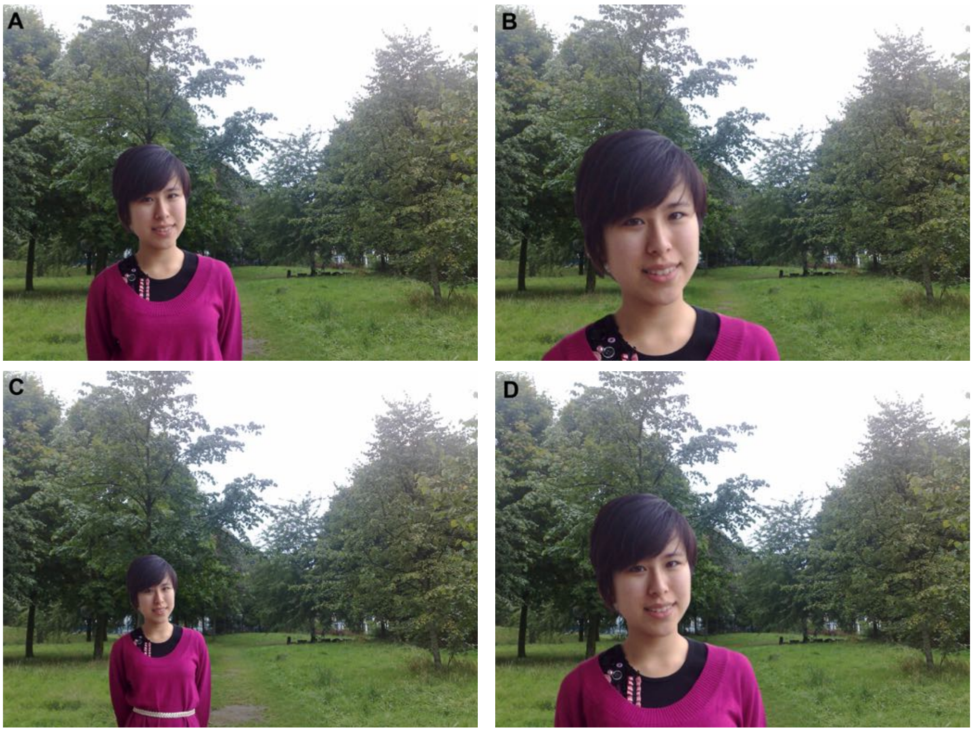

Which picture do you prefer? A/B/C/D

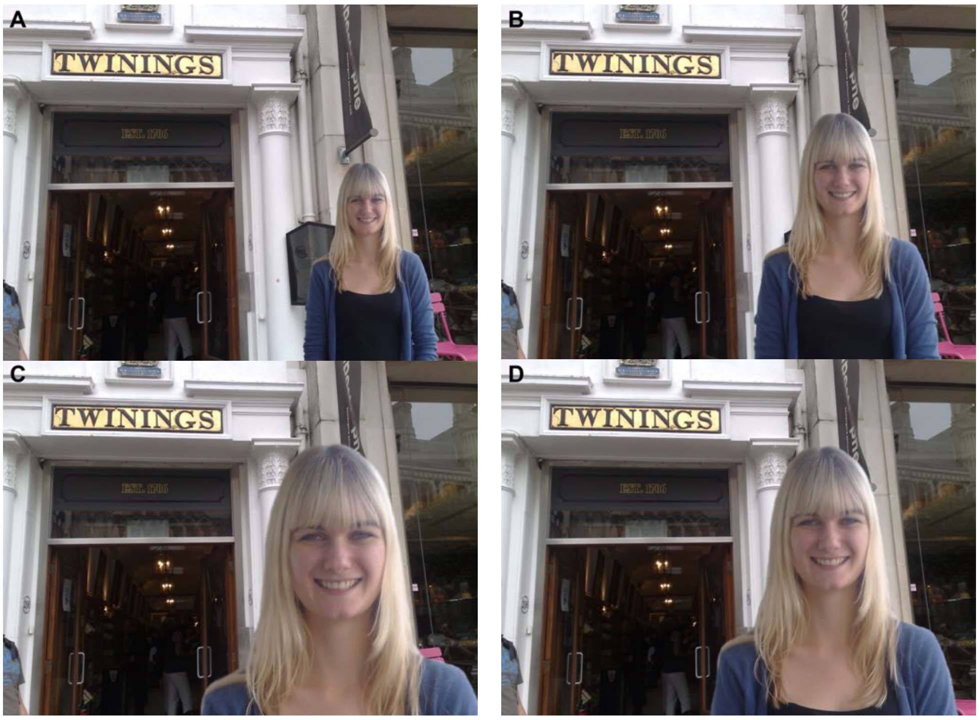

Which picture do you prefer? A/B/C/D

3. Please think of a recent social occasion which you enjoyed, and describe what happened in five sentences.

Example:

1. Last weekend my friend and I decided to cook a meal.
2. We chose a recipe that I didn't know.
3. It took us ages to find the right ingredients for our meal.
4. We forgot to buy sugar, so we used sweeteners instead.
5. I thought the dish was great, but my friend said it was terrible.

1. ____________________

2. ____________________

3. ____________________

4. ____________________

5. ____________________

4. Please mark your answer to the following questions on the scale, for example:

Ex: This instruction is clear

I completely agree | — | I completely disagree

This marking means you think the instruction completely clear.

Please answer the following questions:

1. My personal identity independent of others, is very important to me

I completely agree | — | I completely disagree

2. Having a lively imagination is important to me

I completely agree | — | I completely disagree

3. I will stay in a group if they need me, even when I'm not happy with the group

I completely agree | — | I completely disagree

4. I respect people who are modest about themselves

I completely agree | I completely disagree

5. Even when I strongly disagree with group members, I avoid an argument

I completely agree | I completely disagree

6. It is important to me to respect decisions made by the group

I completely agree | I completely disagree

7. I feel comfortable using someone's first name soon after I meet them, even when they are much older than I am

I completely agree | I completely disagree

8. I am the same person at home that I am at school

I completely agree | I completely disagree

5. On the next page, please draw a landscape including at least a barn, a tree, a cow, a road, and a horizon. You can draw more things if you want to. Please keep the paper horizontal with the arrow pointing down.

Barn, tree, cow, road, horizon

Finally, please answer the following questions about yourself:

1. What is your age? ____________________

2. What is your sex? ____________________

3. In which country were you born? ______________________

4. In which country (or countries) were your parents born?

   Mother: _________________ Father: __________________

5. If you have lived in more than one country in your lifetime, please list those countries and give the age you were when you moved to each country

   ____

6. What is your current occupation? (If you are retired, please give your most recent occupation at the time of retirement)

   ____

7. What are your parents' current or (if retired) most recent occupations?

   Mother: ___________________ Father: _________________

Please stop here and wait for instructions on how to fold your shape.

Tasks v.1: Paper Folding

Part 2:

1. Which of the words belongs most to the first word? Please circle your choice.

	Choose between...	
**Pigeon**	Duck	Nest
**Door**	Key	Window
**Monkey**	Panda	Banana
**Coat hanger**	Dress	Hook
**Cot**	Bed	Baby
**Car**	Garage	Bicycle
**Bee**	Flower	Ant
**Shampoo**	Hair	Conditioner
**Pen**	Paper	Pencil
**Glove**	Scarf	Hand

2. Please indicate which of each four pictures you like best.

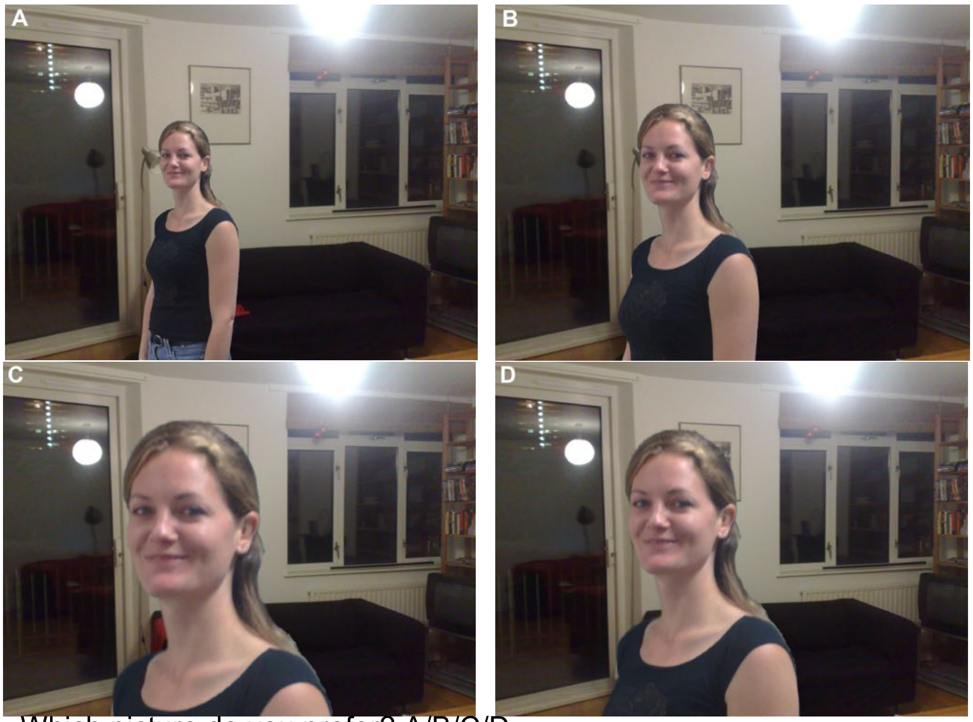

Which picture do you prefer? A/B/C/D

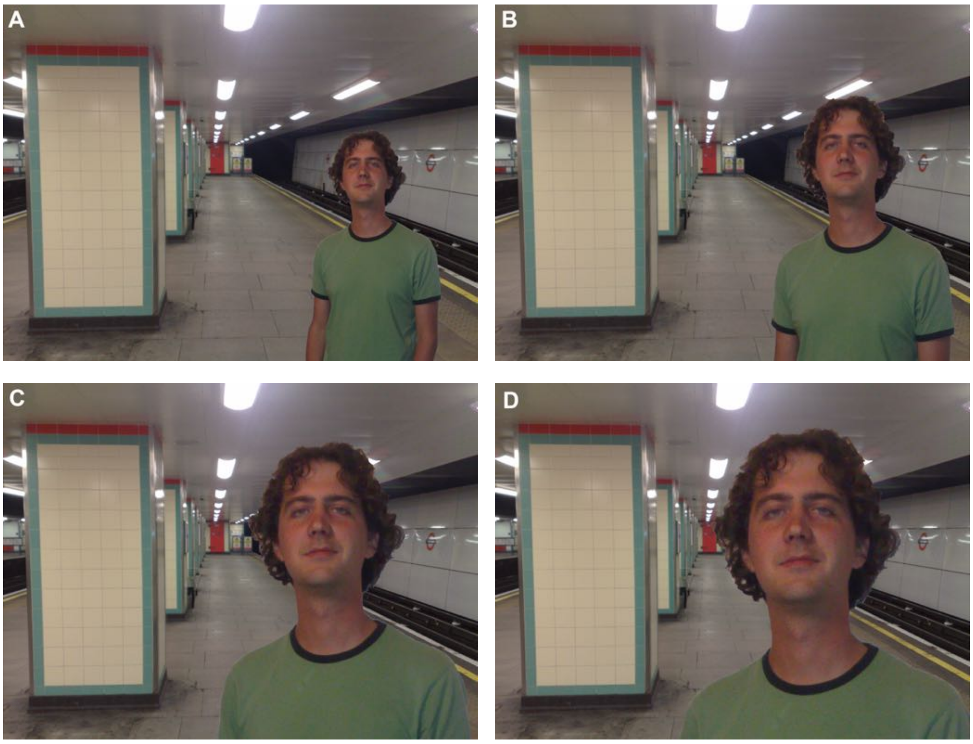

Which picture do you prefer? A/B/C/D

Tasks v.1: Paper Folding

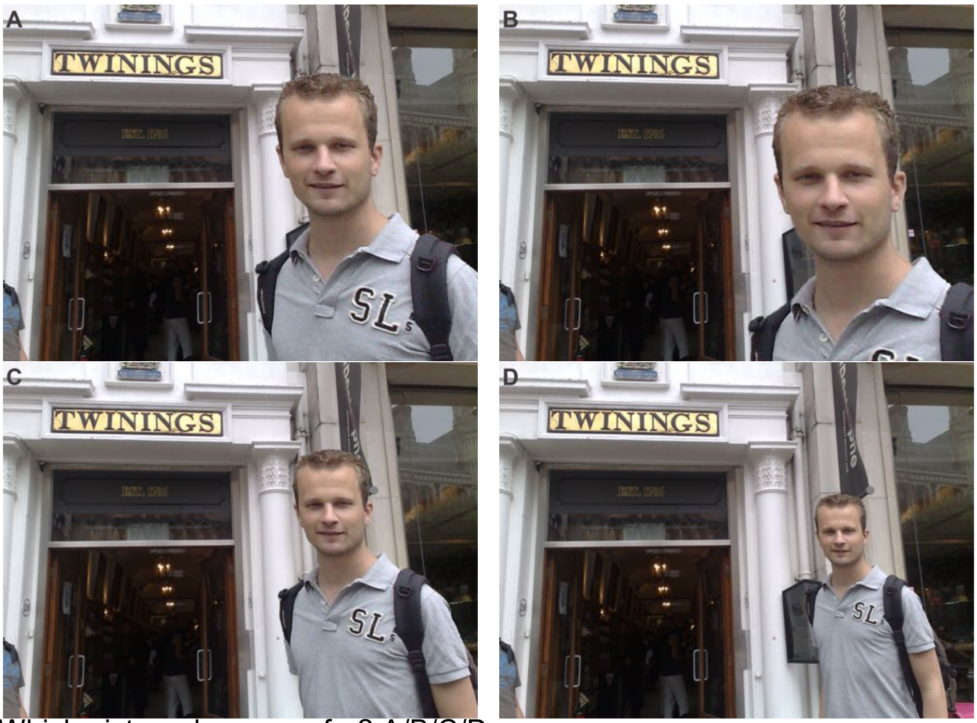

Which picture do you prefer? A/B/C/D

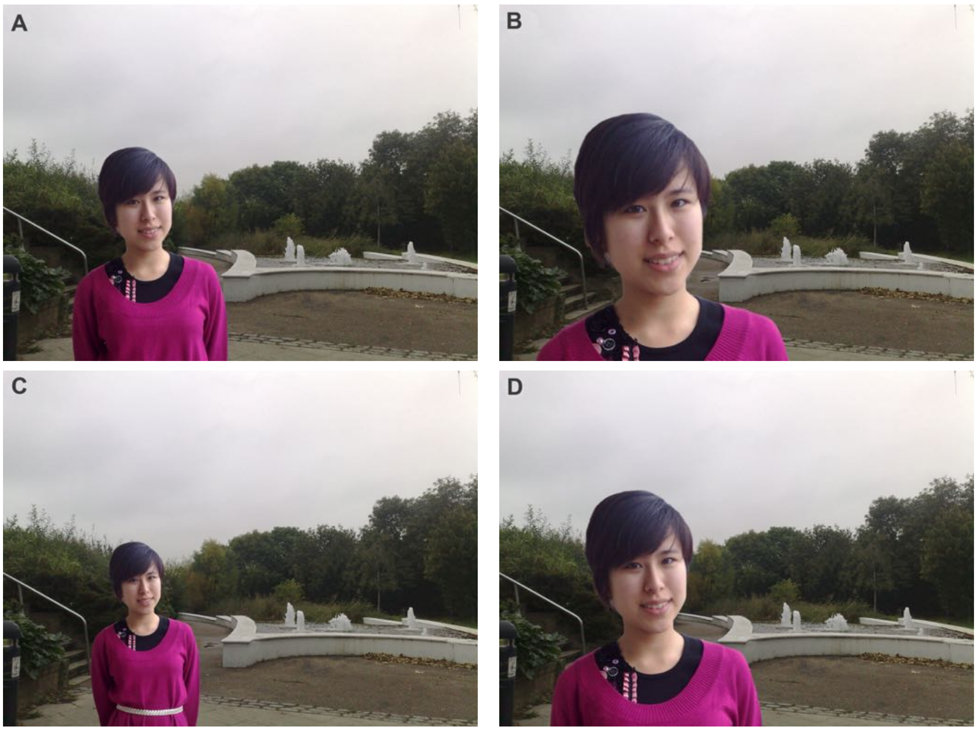

Which picture do you prefer? A/B/C/D

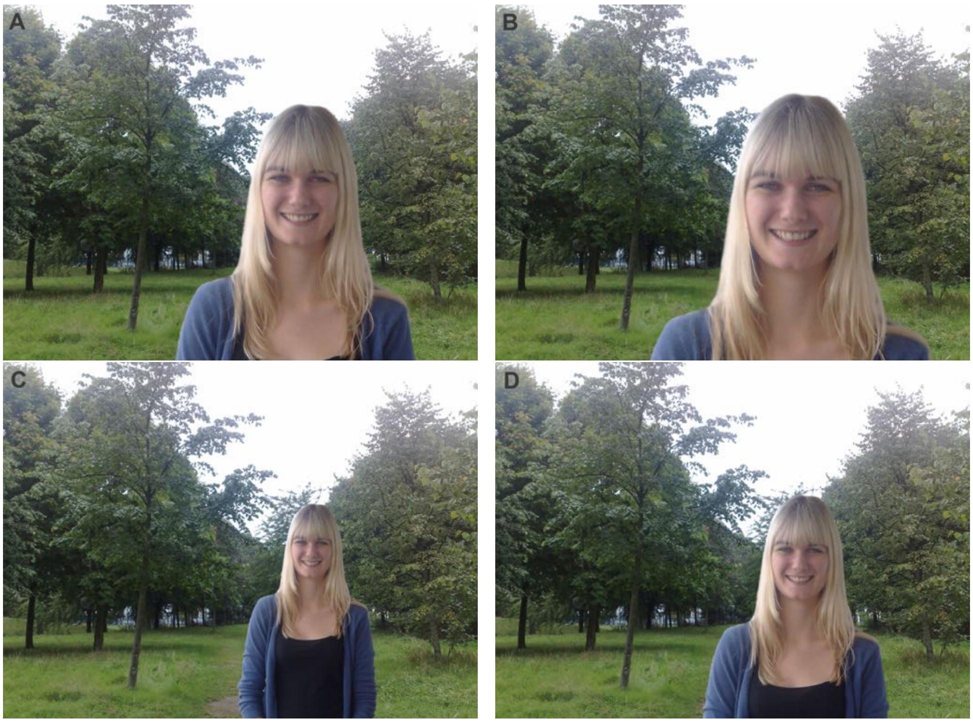

Which picture do you prefer? A/B/C/D

3. Please think of a recent social occasion which you enjoyed, and describe what happened in five sentences.

Example:

1 There was a movie that my friends and I wanted to see for some time.
2 Last Sunday we went, and I got us all the tickets.
3 We went for dinner before the movie.
4 As we expected the movie was great and we had a good time.
5 Because I had class the next morning I went home early.

1. ____
2. ____
3. ____
4. ____
5. ____

Tasks v.1: Paper Folding

4. Please mark your answer to the following questions on the scale, for example:

Ex: This instruction is clear

I completely agree | I completely disagree

This marking means you think the instruction completely clear.

Please answer the following questions:

1. If my brother or sister fails, I feel responsible

I completely agree | I completely disagree

2. I often have the feeling that my relationships with others are more important than my own accomplishments

I completely agree | I completely disagree

3. Being able to take care of myself is a primary concern for me

I completely agree | I completely disagree

4. I prefer to be direct and forthright when dealing with people I've just met

I completely agree | —————————— | **I completely disagree**

5. I enjoy being unique and different from others in many respects

I completely agree | —————————— | **I completely disagree**

6. I should take into consideration my parents' advice when making education/career plans

I completely agree | —————————— | **I completely disagree**

7. My happiness depends on the happiness of those around me

I completely agree | —————————— | **I completely disagree**

8. I am comfortable with being singled out for praise or rewards

I completely agree | —————————— | **I completely disagree**

5. On the next page, please draw a landscape including at least a house, a tree, a river, a person, and a horizon. You can draw more things if you want to. Please keep the paper horizontal with the arrow pointing down.

House, tree, river, person, horizon
