# Supplementary material for: Experimental priming of independent and interdependent activity does not affect culturally variable psychological processes
Source: R Soc Open Sci. 2017 May 17;4(5):161025. doi: 10.1098/rsos.161025 (PMC5451795; doi:10.1098/rsos.161025)
Supplement: File S6 [file rsos161025supp8.pdf]

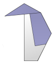

Penguin

Step 1. Turn paper so it's a diamond shape and white side up in front of you, then fold across the middle to make a triangle.	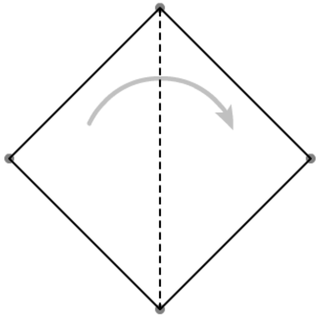
Step 2. Fold again from the bottom corner to go halfway across the triangle.	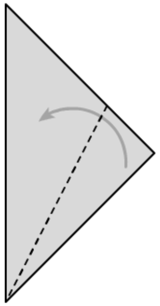
Step 3. Fold over a small triangle across the top right corner, then unfold it back out.	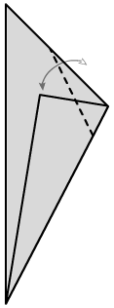

Step 4. Now unfold and open out all the folds of the paper	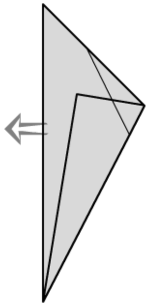
Step 5. With the white side up, fold both side corners inward along the previous crease.	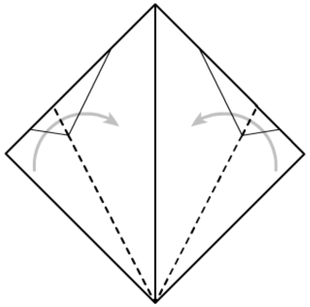
Step 6. Fold the side corners **underneath** along the crease.	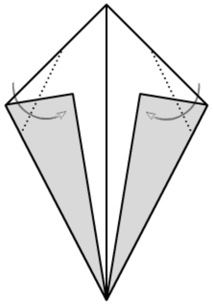

Step 7. Turn the paper over to the coloured side and put the smallest corner pointed up	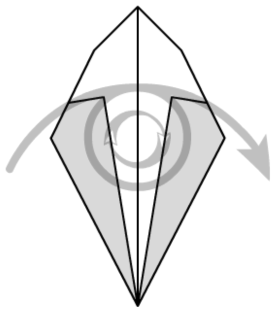
Step 8. Fold the bottom corner over up to where the two folds on the sides begin	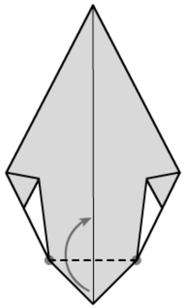
Step 9. Fold inward along the middle crease	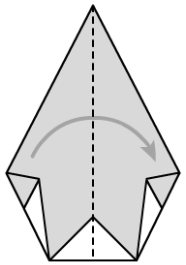

Step 10. Fold the top corner over diagonally.	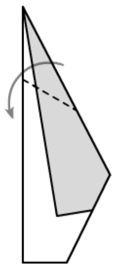
Step 11. Unfold the top corner from step 10.	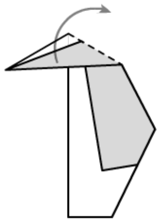
Step 12. Fold the top corner outward over both sides along the previous crease.	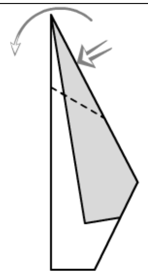
Your finished penguin should look something like this!	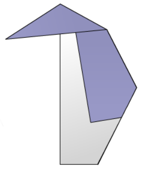

Wriggly Fish

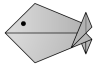

Step 1. Turn paper so it's a square shape and white side up in front of you, then fold in half diagonally to make a triangle and open it back out. Repeat to make another diagonal crease.	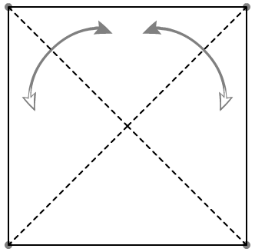
Step 2. From one corner of the square fold two triangles half way across the paper up to the crease and fold back out. Do the same thing from the diagonally opposite as well.	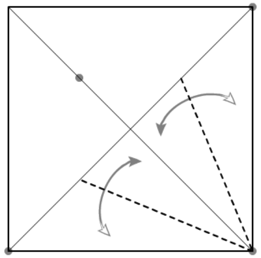
Step 3. Fold outward from the small dotted line at the middle and inward along the long folds.	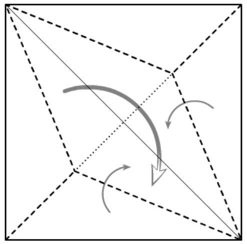
Step 4. Fold diagonally and upward from the smallest corner of the paper. Then turn the paper over and repeat for the other side.	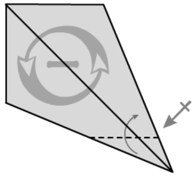
Step 5. Move the fins back and forth to make the fish wriggle.	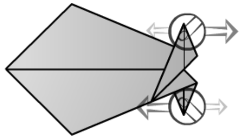
At the end, your Wriggly Fish should look something like this!	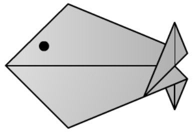

Piano

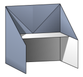

Step 1. Turn paper so it’s a square shape and white side up in front of you, then fold in half across the middle and open back out.	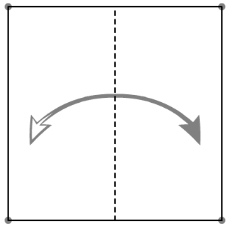
Step 2. Fold over across the middle from the top	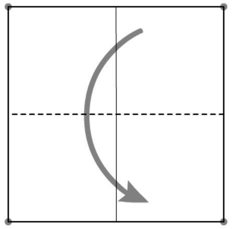
Step 3. Fold across to the middle crease on both sides	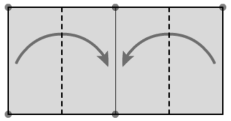
Step 4. Fold diagonally outward from the middle of the rectangle, then unfold.	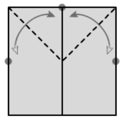

Step 5. Fold outward from the diagonal crease while opening out the sides completely.	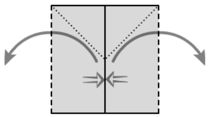
Step 6. Fold the middle piece of paper upward at the halfway crease.	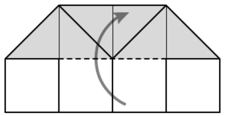
Step 7. Fold the rectangle in half down to the middle crease, then unfold it.	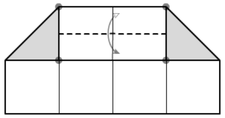
Step 8. Fold the top quarter of the middle rectangle down to the halfway crease.	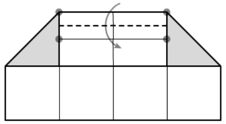
Step 9. Fold again at the crease at the top of the centre rectangle	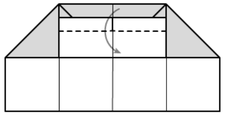

Step 10. Fold both sides inward	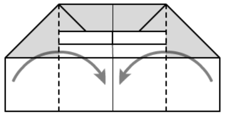
Step 11. Pull the central rectangle back down and fold the two sides outward halfway	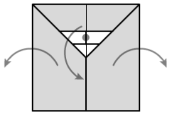
At the end, your piano should look something like this!	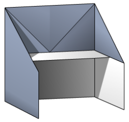

Swan *

1.

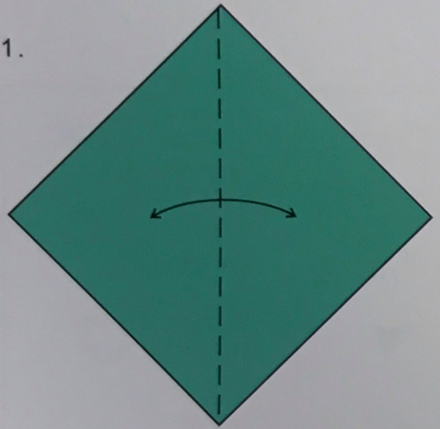

2.

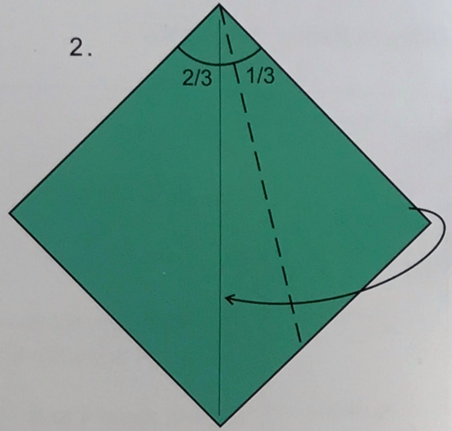

3.

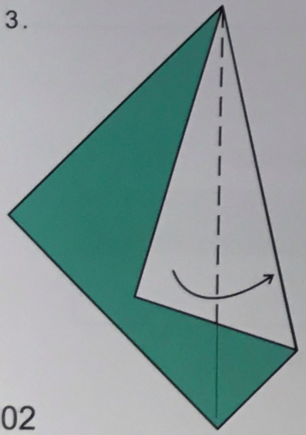

4.

(2 to 3)

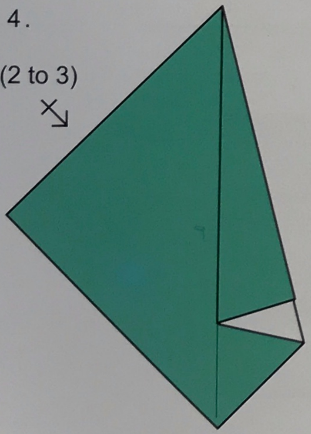

5.

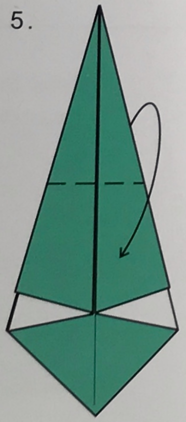

6.

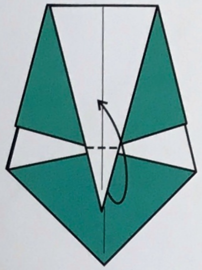

7.

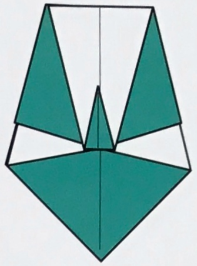

turn over

8.

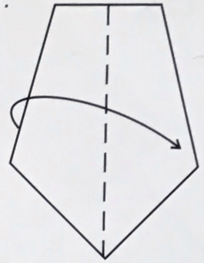

9.

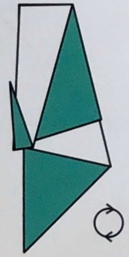

90°

10.

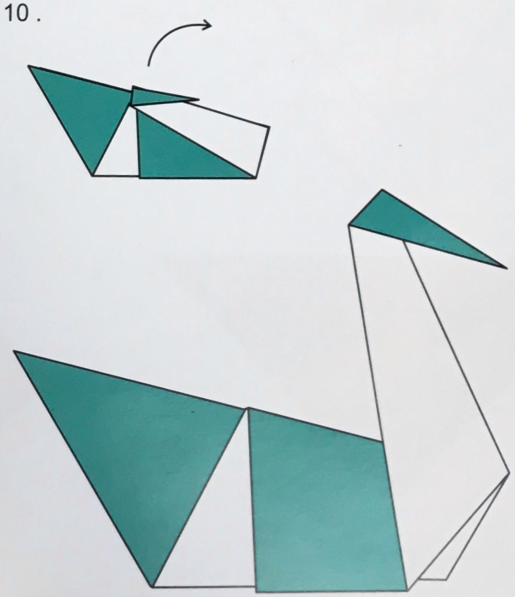

Boat (traditional)

Diagrammed by: František Grebeníček (1999)
www.origami.cz

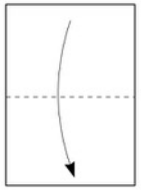

(1) Start from a rectangle (e.g. A4). Fold in half.

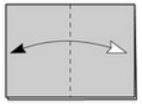

(2) Fold in half and unfold.

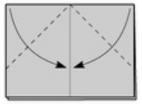

(3) Fold to the center.

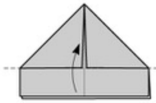

(4) Fold the overlapping strip upwards.

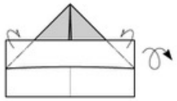

(5) Fold corners backwards. Turn over.

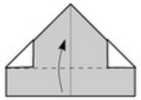

(6) Fold strip upwards.

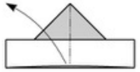

(7) Open.

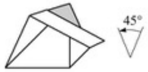

(8) Opening in progress.

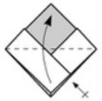

(9) Fold triangle upwards. Repeat behind.

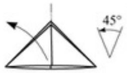

(10) Open (like in the steps 7 and 8).

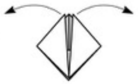

(11) Take upper corners and stretch out.

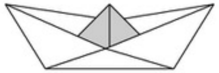

(12) Finished boat.
